# Examining Twitter-Derived Negative Racial Sentiment as Indicators of Cultural Racism: Observational Associations With Preterm Birth and Low Birth Weight Among a Multiracial Sample of Mothers, 2011-2021

**DOI:** 10.2196/44990

**Published:** 2023-04-28

**Authors:** Thu T Nguyen, Junaid S Merchant, Shaniece Criss, Katrina Makres, Krishik N Gowda, Heran Mane, Xiaohe Yue, Yulin Hswen, M Maria Glymour, Quynh C Nguyen, Amani M Allen

**Affiliations:** 1 Department of Epidemiology & Biostatistics University of Maryland School of Public Health College Park, MD United States; 2 Department of Health Sciences Furman University Greenville, SC United States; 3 Department of Epidemiology and Biostatistics Bakar Computational Health Sciences Institute University of California, San Francisco San Francisco, CA United States; 4 Department of Epidemiology and Biostatistics University of California, San Francisco San Francisco, CA United States; 5 Divisions of Community Health Sciences and Epidemiology University of California, Berkeley Berkeley, CA United States

**Keywords:** birth outcomes, health disparities, machine learning, racial sentiment, social media

## Abstract

**Background:**

Large racial and ethnic disparities in adverse birth outcomes persist. Increasing evidence points to the potential role of racism in creating and perpetuating these disparities. Valid measures of area-level racial attitudes and bias remain elusive, but capture an important and underexplored form of racism that may help explain these disparities. Cultural values and attitudes expressed through social media reflect and shape public norms and subsequent behaviors. Few studies have quantified attitudes toward different racial groups using social media with the aim of examining associations with birth outcomes.

**Objective:**

We used Twitter data to measure state-level racial sentiments and investigate associations with preterm birth (PTB) and low birth weight (LBW) in a multiracial or ethnic sample of mothers in the United States.

**Methods:**

A random 1% sample of publicly available tweets from January 1, 2011, to December 31, 2021, was collected using Twitter’s Academic Application Programming Interface (N=56,400,097). Analyses were on English-language tweets from the United States that used one or more race-related keywords. We assessed the sentiment of each tweet using support vector machine, a supervised machine learning model. We used 5-fold cross-validation to assess model performance and achieved high accuracy for negative sentiment classification (91%) and a high F1 score (84%). For each year, the state-level racial sentiment was merged with birth data during that year (~3 million births per year). We estimated incidence ratios for LBW and PTB using log binomial regression models, among all mothers, Black mothers, racially minoritized mothers (Asian, Black, or Latina mothers), and White mothers. Models were controlled for individual-level maternal characteristics and state-level demographics.

**Results:**

Mothers living in states in the highest tertile of negative racial sentiment for tweets referencing racial and ethnic minoritized groups had an 8% higher (95% CI 3%-13%) incidence of LBW and 5% higher (95% CI 0%-11%) incidence of PTB compared to mothers living in the lowest tertile. Negative racial sentiment referencing racially minoritized groups was associated with adverse birth outcomes in the total population, among minoritized mothers, and White mothers. Black mothers living in states in the highest tertile of negative Black sentiment had 6% (95% CI 1%-11%) and 7% (95% CI 2%-13%) higher incidence of LBW and PTB, respectively, compared to mothers living in the lowest tertile. Negative Latinx sentiment was associated with a 6% (95% CI 1%-11%) and 3% (95% CI 0%-6%) higher incidence of LBW and PTB among Latina mothers, respectively.

**Conclusions:**

Twitter-derived negative state-level racial sentiment toward racially minoritized groups was associated with a higher risk of adverse birth outcomes among the total population and racially minoritized groups. Policies and supports establishing an inclusive environment accepting of all races and cultures may decrease the overall risk of adverse birth outcomes and reduce racial birth outcome disparities.

## Introduction

Low birth weight (LBW) and preterm birth (PTB) are widely used indicators of reproductive health [1,2] and are associated with an increased risk of infant mortality [3], developmental delays [4], and cardiometabolic disorders in adulthood [5,6]. Large racial disparities in these birth outcomes persist in the United States [7,8]. Black mothers in particular have substantially higher rates of LBW and PTB, infant mortality, and maternal morbidity compared to White mothers. For example, pregnancy-related mortality is over 3 times higher among Black compared to White women, and LBW rates in 2020 were 14.2% for Black mothers, but 6.8% for White mothers [7]. These disparities cannot be fully accounted for by sociodemographic and individual-level factors [7-9], and there is increasing evidence for the role of racism in creating and perpetuating race-based disparities in birth outcomes [10,11].

Racism is a well-established social determinant of health that operates at and across multiple levels. This includes the internalized, personally mediated (also referred to as individual or interpersonal), institutional, structural, and cultural dimensions of racism [12]. Internalized racism refers to the acceptance of negative beliefs about their own race by individuals of racially stigmatized groups [13]. Personally mediated racism refers to racial prejudice (attitudes) and stereotypes (beliefs and assumptions) according to race and discrimination (differential treatment based on race) enacted between individuals [14]. Institutional racism refers to laws, policies, and practices of particular institutions in providing advantages and disadvantages, differentially, according to race [15]. Structural racism involves the coordination and interaction of multiple institutions and systems, such as those involved in housing, prison, banking, and education, to provide differential access and resources according to racial group identity [16]. Research has found experiences of racism to be associated with a wide variety of health outcomes [17,18]. However, most of this research has examined how racism operates at the individual level, whereas work investigating how racism operates at other levels has been relatively limited.

Exposure to racism is also a psychosocial stressor that, when experienced chronically, has demonstrated negative health effects that contribute to birth outcome disparities [11,19]. Maternal stress can alter neuroendocrine function, impact immune and inflammatory responses, and affect the vascular system. Racism stress, in particular, is associated with increases in the systemic corticotropin-releasing hormone, which stimulates the release of prostaglandins from the placenta and facilitates oxytocin’s role in initiating contractions earlier in pregnancy [20]. Stress can prompt vasoconstriction, increasing blood pressure and reducing blood flow to the fetus [21]. Stress also impacts the immune system, hindering its ability to fight infections that linger in the body longer, and is associated with an increased likelihood of preterm labor [21]. Racism-related stress can also lead to maladaptive coping behaviors, such as smoking and alcohol use. Additionally, women who are born with LBW are more likely to give birth to children with LBWs [19], perpetuating the impacts of racism intergenerationally. Racism may also influence birth outcomes by limiting access to resources and opportunities, such as education, employment, health care, and housing [16,17]. For example, historical practices like redlining and discriminatory home loan lending led to racial residential segregation and disinvestment in communities of color, which continue to have measurable effects on health disparities today, including racial birth outcome disparities [22].

An emerging body of research has revealed the foundational role of cultural racism in perpetuating race-based disparities. Cultural racism is the infusion of the ideology of racial hierarchy into the values, language, imagery, symbols, and unstated assumptions of the larger society [17]. It is displayed through the media, stereotyping, and norms within society and its institutions [23]. Importantly, cultural racism produces a context that supports the other dimensions of racism to maintain and reinforce health inequities [17]. Cultural racism, or the accepted norms, values, and ideologies of a racialized society, becomes realized in policies and practices within and across institutions [24]. In this way, cultural and structural racism are mutually reinforcing. One way of tapping into cultural racism is to assess community members’ attitudes toward other racial groups [25,26]. A promising line of work has used such approaches to assess the impact of area-level measures of racial prejudice (also referred to as sentiment or animus) on health. A 2022 systematic review of the literature on area-level prejudice and health revealed that all studies found an association between higher area-level prejudice and worse health outcomes among racially minoritized groups, and 4 studies even showed the negative impact of prejudice among White samples [27]. However, research on birth outcomes is limited as only 4 of the studies in the aforementioned review investigated birth outcomes [27]. Moreover, measuring racial sentiment using traditional survey approaches can be costly, time-consuming, and subject to self-report biases [28-30]. Alternatively, social media data provide unique opportunities for assessing population-level racial sentiment, which can be used as an indicator of cultural racism.

Twitter represents a rapid mode of communication, with millions of tweets sent daily by users across the globe, 80%-90% of whom have publicly accessible Twitter profile [31]. Cultural values and attitudes expressed on the web through social media both reflect and shape public norms, beliefs, and subsequent behaviors [32,33]. Few studies have attempted to quantify attitudes toward different racial groups using Twitter data with the aim of examining how it relates to birth outcomes. Using state-level data from 2015 to 2017, our previous study demonstrated that state-level racial sentiment was associated with implicit and explicit racial bias [10], and exhibited associations with birth outcomes of different groups of minority women [34]. This study aims to extend the findings of previous work by (1) examining a wider time frame (2011-2021) to assess whether the associations between Twitter-derived racial sentiment and birth outcomes are persistent across the years, and (2) through a closer examination of the distribution of attitudes toward different racial groups at the state- *and* county-level to see how they related to birth outcomes within and across racial or ethnic groups.

## Methods

A random sample comprising 1% of publicly available tweets from January 1, 2011 to December 31, 2021 was collected using Twitter’s Academic Application Programming Interface. We restricted our analyses to tweets that were in English, from the United States, and used one or more race-related keywords (Multimedia Appendix 1, Table S1). These race-related keywords were constructed based racial and ethnic terms from the US Census, previous studies examining race-related web-based conversations [35], and a web-based database of racial slurs [36]. We included tweets with a unique tweet id. We dropped duplicate tweets according to their “tweet_id.” Retweets and quoted tweets are included in this data. Our analytic sample included 56,400,097 tweets from 3,699,646 unique users.

### Sentiment Analysis

We assessed the sentiment of each tweet using a support vector machine (SVM), a supervised machine learning model. A full description of our model has been previously described by Nguyen and colleagues [34]. Our training data was comprised of manually labeled tweets obtained from Sentiment140 (n=498) [37], Kaggle (n=7086) [38], Sanders (n=5113) [39], as well as 6481 tweets labeled by our research group. Sentiment140, Sanders, and Kaggle datasets are all publicly available training datasets specifically labeled for sentiment analysis. We used 5-fold cross-validation to assess model performance and achieved a high level of accuracy for the negative sentiment classification (91%) and a high F1 score (84%). Accuracy is measured as the number of posts with the correct prediction divided by the total number of tweets in the testing data set. The F1 score is a measure that balances precision (positive predicted value and recall [sensitivity]); a high F1 score suggests a model is robust in predicting posts that are labeled as 1. The trained SVM model was used to analyze our Twitter data set for negative sentiment classification. Please see the code for the data collection and sentiment analysis model in Multimedia Appendix 1.

To assess historical trends in sentiment, average negative sentiment scores for each racial and ethnic group along with the sentiment scores referencing racially minoritized groups were plotted as a function of time. The line chart was plotted using the “Matplotlib” library in Python [40]. We visualized the average negative sentiment scores with geographic plots using the Tableau software. The sentiment scores were obtained from the SVM model and grouped using the state Federal Information Processing Standards (FIPS) codes.

### Individual-Level Birth Outcomes Data

Individual birth outcomes data through 2011-2021 were obtained from restricted US natality files that included geographic identifiers. The latest year the natality files are currently available is 2021. We chose 2011 to examine trends in the last decade. In addition, Twitter introduced Twitter “Places” for geotagging tweets in June 2010 [41]. The natality files include all births in the United States during this time period. The data come from birth certificates filed in each state. The analysis was restricted to singleton births with no congenital abnormalities. These exclusion criteria helped ensure that associations with our birth outcomes were not due to congenital abnormalities [42] or twins, triplets, and other higher order multiple births, which are known to increase the risk for LBW and PTB [43]. The primary outcomes were LBW (defined as birth weight <2499 g) and PTB (defined as gestational age <37 weeks).

### Covariates

We adjusted for potential confounders at the individual- and area-level when assessing the association between racial sentiment and birth outcomes. Individual-level covariates in our models included birth year, maternal age (linear spline with knots at 19, 25, 29, 33, and 38 years), race (non-Hispanic White, non-Hispanic Black, non-Hispanic Asian), Hispanic ethnicity, marital status (married or unmarried), education (less than high school, high school or General Education Development, some college, bachelor’s degree, master’s degree, or doctorate), and first birth (yes or no), and prenatal care initiation during the first trimester (yes or no). We also adjusted for state-level characteristics, including proportions of non-Hispanic Black and Latinx individuals, population density (per square mile), and economic disadvantage (standardized factor score) [44,45] summarizing the following variables (%): unemployed; some college education, high school diploma, children in poverty, single parent household, and median household income) to account for state-level compositional differences in demographic and economic characteristics. Use of the factor score has been previously established [46]. State-level covariates were derived from 2011 to 2021 from the American Community Survey [47].

### Statistical Analyses

For each year, the state-level racial sentiment was merged with birth data during that year. The racial sentiment was coded in tertiles for analysis assessing associations with birth outcomes. We estimated incidence ratios for LBW and PTB (separately) using log binomial regression models with state-level racial sentiment as our independent variable of interest, and controlled for individual-level maternal characteristics and state-level demographic characteristics. We evaluated statistical significance at *P*<.05. Our primary models examined the association between state-level sentiment toward all racially minoritized groups on birth outcomes among the full sample, racially minoritized mothers, and White mothers. Follow-up analyses examined associations between state-level sentiment toward individual racial groups (Asian, Black, Latinx, and White) and birth outcomes for those groups specifically. Sensitivity analyses were conducted to examine the association between county-level racial sentiment and birth outcomes. Although all tweets collected had place information that permitted the identification of the state, only 60% (n=33,840,058) of tweets collected had place information to identify the county. Thus, we consider the county-level analyses to be supplementary. Sensitivity analyses also excluded tweets from users who tweeted more than 1000 times per year, which represented less than 1% (n=1066) of all tweets. Stata MP 16 [48] was used for statistical analysis.

### Ethical Considerations

This study was determined exempt by the University of Maryland College Park Institutional Review Board (1797788-1).

## Results

**Figure 1:**
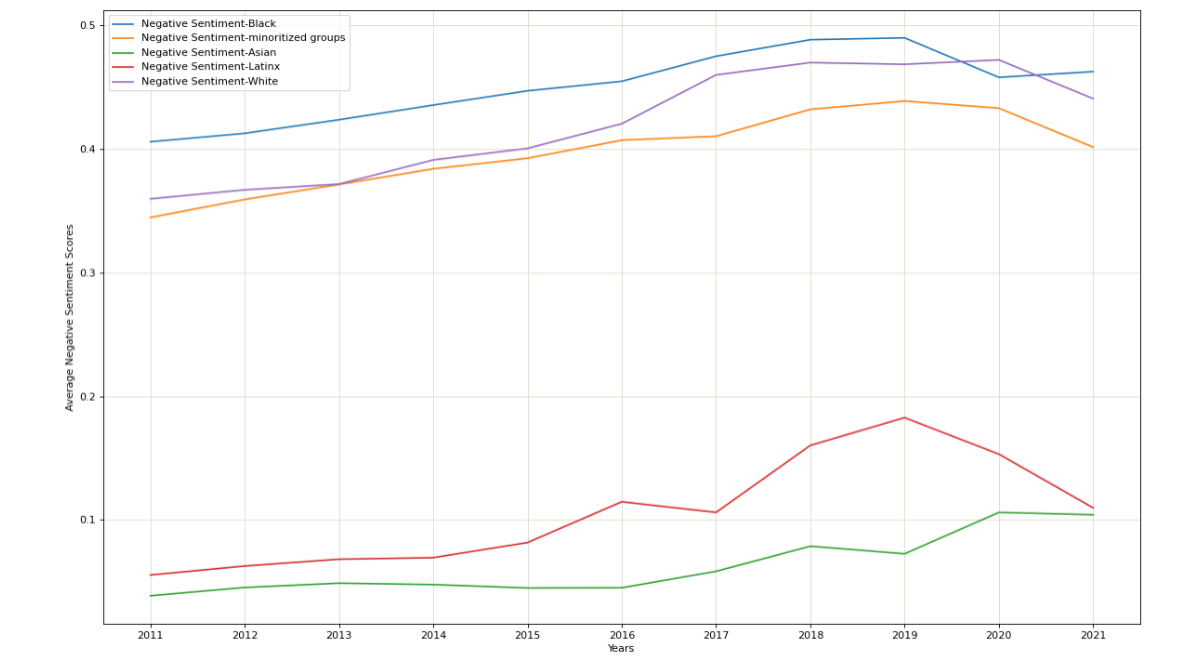
Temporal trends in proportion of tweets that were negative by racial and ethnic group. Data source: Twitter data from 2011 to 2021.

Among the terms assessed, the top 20 terms by year were present in 86% (n=48,374,805) of all tweets concerning a racial or ethnic minority group (Multimedia Appendix 1, Table S2). The proportion of tweets referencing Black people, Latinx, and White people that were negative increased over time, peaking in 2019. For tweets referencing White people and Latinx, we saw a plateauing or declining trend in 2020-2021. The proportion of tweets referencing Asians that were negative climbed until 2020 and declined slightly in 2021 (Figure 1). Black people experienced the highest negative sentiment of all groups. After a brief period of decline in 2020, the proportion of tweets referencing Black people that were negative began to rise again. Higher proportions of negative tweets referencing Black Americans were found in the Southern and Northeastern US regions. Geographic distribution of tweets referencing racially minoritized groups, Latinx, and Asians are presented in Multimedia Appendix 1 (Figures S1-3).

Approximately 55% of births were to White mothers; 22% of the samples were Latinx. Black and Asian mothers made up 15% (n=5,152,595) and 6% (n=2,116,785) of the sample, respectively. The majority of women were US born (78%, n=27,630,726), and 31% (n=11,088,533) completed college or beyond.

**Figure 2:**
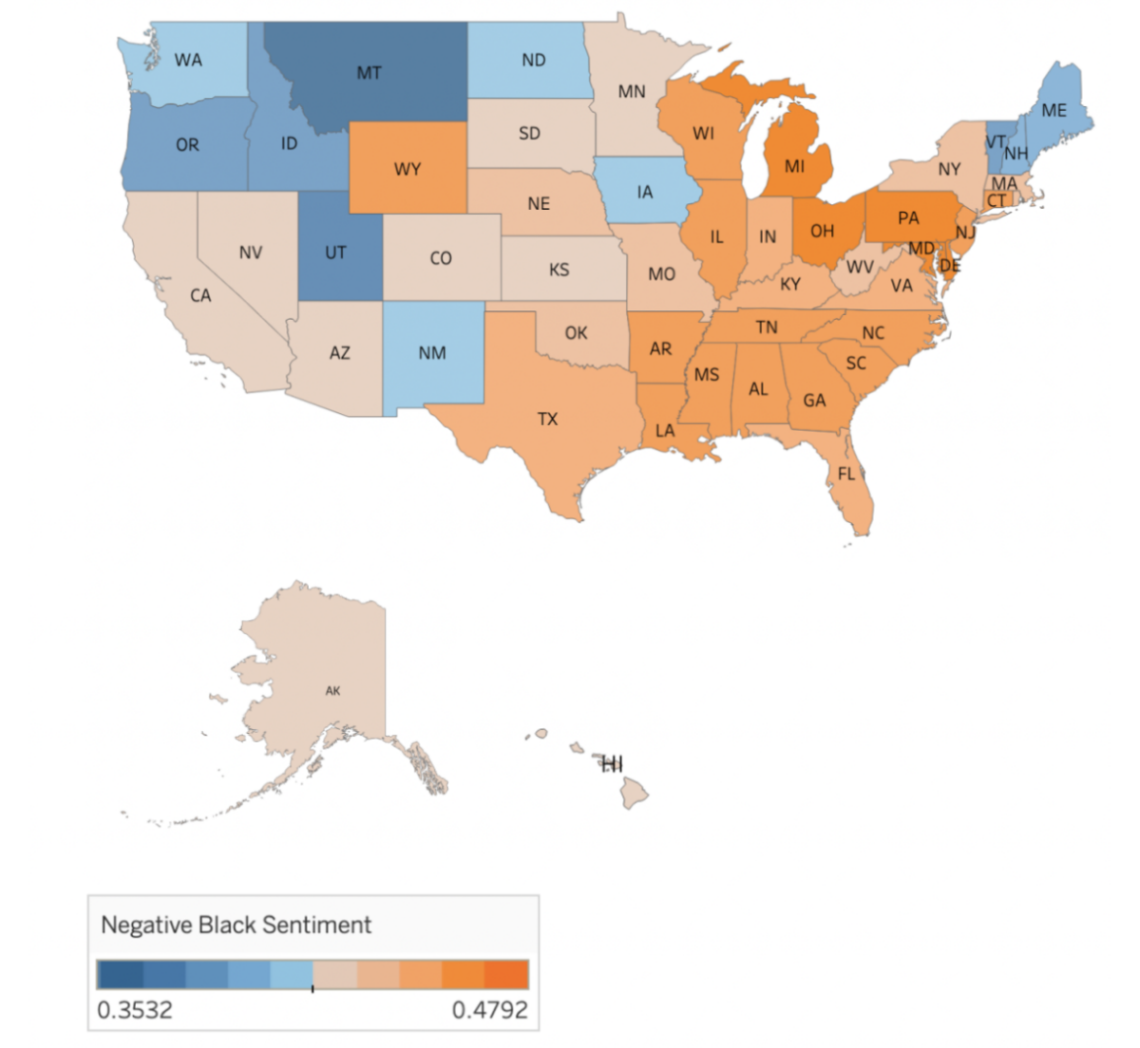
Geographic distribution of averaged negative sentiment of tweets referencing Black people. Data source: Twitter data from 2011 to 2021.

The state-level negative racial sentiment was consistently associated with adverse birth outcomes controlling for individual maternal characteristics and state-level sociodemographic characteristics (Tables 1 and 2). For the total population, mothers living in states in the highest (third) tertile of negative racial sentiment for tweets referencing racial and ethnic minoritized groups have an 8% higher (95% CI 3%-13%) incidence of LBW and a 5% higher (95% CI 0%-11%) incidence of PTB compared to mothers living in the lowest (first) tertile of negative racial sentiment. Moreover, associations were found for mothers living in states in the second vs lowest tertile of negative racial sentiment. Negative racial sentiment referencing racially minoritized groups was associated with birth outcomes in the total population, among minoritized mothers and White mothers. Associations are slightly higher for racially minoritized mothers in all but one of the models where racially minoritized and White mothers show similar associations with racial sentiment (Figure 2).

When examining race- and ethnic-specific sentiment and birth outcomes, the strongest and most consistent associations were found for negative sentiment in tweets referencing Black people and birth outcomes among Black mothers. Black mothers living in states in the third tertile of negative Black sentiment had a 6% higher incidence of LBW and a 7% higher incidence of PTB compared to mothers living in the lowest tertile states. Negative sentiment in tweets referencing Latinx individuals was associated with a 6% and 3% higher incidence of LBW and PTB, respectively, comparing Latinx mothers living in the highest compared to the lowest tertile states. Negative sentiments referencing White individuals was not associated with birth outcomes among White mothers (Table 3).

Multimedia Appendix 1 tables present these associations by year (Tables S3-6). Associations between state-level negative racial sentiment for tweets referencing minoritized groups tended to be more strongly associated with birth outcomes among minoritized mothers compared to the total population of birthing people or of White mothers. There were small fluctuations in estimates across the years. The overall negative state-level racial sentiment was associated with adverse birth outcomes for most years. Negative sentiment referencing Black Americans was consistently associated with an elevated risk of LBW and PTB among Black mothers. Negative sentiment in tweets referencing Latinx and Asian individuals was associated with adverse birth outcomes among Latinx and Asian mothers for selected years.

Sensitivity analyses excluded tweets from users who tweeted more than 1000 per year. The estimates changed slightly, but all main conclusions remain the same (Multimedia Appendix 1, Tables S7 and S8). Sensitivity analyses examining the associations between county-level racial sentiment and birth outcomes (Multimedia Appendix 1, Tables S9-14) found attenuated associations compared to state-level analyses. The restricted natality files only included state- or county-geographic identifiers, so more granular analyses were not possible with that data. Compared to state-level analyses, small increases in risks of PTB and LBW among minoritized women were observed when comparing mothers living in the highest versus lowest tertile counties (see combined county-level in Multimedia Appendix 1, Tables S6-7).

**Table 1:** Characteristics of mothers giving birth from 2011 to 2021 (N=35,267,177).

Characteristic		Value^a^
Age (years), mean (SD)		28.50 (5.85)
Married, n (%)		21,068,841 (59.74)
White, n (%)		19,236,693 (54.55)
Black, n (%)		5,152,595 (14.61)
Asian, n (%)		2,116,785 (6.00)
Latinx, n (%)		7,932,703 (22.49)
US born, n (%)		27,630,726 (78.35)
**Education, n (%)**		
	Less than high school	5,018,290 (14.23)
	High school	8,999,298 (25.61)
	Some college	10,161,059 (28.81)
	College	7,014,807 (19.89)
	Master’s or doctorate	4,073,726 (11.55)
First birth, n (%)		11,318,052 (32.09)
Prenatal care during first trimester, n (%)		26,832,305 (76.09)
**Birth outcomes, n (%)**		
	Low birth weight	2,215,586 (6.28)
	Preterm birth	2,770,462 (7.86)

^a^Data source: US natality files from 2011 to 2021.

**Table 2:** Associations using incidence rate ratios between state-level racial sentiment toward minoritized and birth outcomes for full sample, minoritized mothers, and White mothers, 2011-2021.^a^

Data		Total, incidence rate ratio (95% CI)	Minoritized groups, incidence rate ratio (95% CI)	White mothers, incidence rate ratio (95% CI)
**Low birth weight**				
	Second tertile vs first (lowest)	1.07 (1.02-1.13)	1.08 (1.02-1.15)	1.07 (1.01-1.13)
	Third tertile vs first (lowest)	1.08 (1.03-1.13)	1.09 (1.03-1.15)	1.08 (1.03-1.14)
	Sample, n	35,267,177	17,190,739	19,236,695
**Preterm**				
	Second tertile vs first (lowest)	1.06 (1.01-1.11)	1.07 (1.02-1.12)	1.05 (1.0-1.11)
	Third tertile vs first (lowest)	1.05 (1.00-1.11)	1.06 (1.01-1.11)	1.06 (0.99-1.13)
	Sample, n	35,288,014	17,200,907	19,247,910

^a^Data source: US natality files and Twitter data from 2011 to 2021.

**Table 3:** Race and ethnic associations using incidence rate ratios between state-level negative sentiment and birth outcomes, 2011-2021.^a^

Data		Negative Black sentiment, birth outcomes among Black mothers, incidence rate ratio (95% CI)	Negative Asian sentiment, birth outcomes among Asian mothers, incidence rate ratio (95% CI)	Negative Latinx sentiment, birth outcomes among Black mothers, incidence rate ratio (95% CI)	Negative White sentiment, birth outcomes among White mothers, incidence rate ratio (95% CI)
**Low birth weight**					
	Second tertile vs first (lowest)	1.08 (1.03-1.13)	0.99 (0.96-1.03)	1.00 (0.97-1.02)	1.00 (0.97-1.04)
	Third tertile vs first (lowest)	1.06 (1.01-1.11)	1.01 (0.96-1.05)	1.06 (1.01-1.11)	1.00 (0.97-1.04)
	Sample, n	5,152,595	2,116,785	7,932,703	19,236,695
**Preterm**					
	Second tertile vs first (lowest)	1.08 (1.03-1.14)	1.01 (0.97-1.05)	0.99 (0.98-1.0)	1.01 (0.98-1.04)
	third tertile vs first (lowest)	1.07 (1.02-1.13)	1.03 (0.99-1.07)	1.03 (1.00-1.06)	0.99 (0.96-1.03)
	Sample, n	5,157,600	2,117,808	7,935,836	19,247,910

^a^Data source: US natality files and Twitter data from 2011 to 2021.

## Discussion

This study used a combination of social media data and comprehensive US birth records to assess how spatial patterns in attitudes toward different racial groups on Twitter related to birth outcomes. We calculated negative sentiment toward different racial groups at the state level and found that negative attitudes toward minoritized groups were associated with increased risk for low birth-weight babies and PTB for the total population, and this remained generally consistent across each of the years we analyzed. Furthermore, we found that negative sentiment toward minoritized groups was comparably associated with birth outcomes for each racial group, including White women when stratifying outcomes by race. In contrast, negative sentiment toward White people was not associated with adverse birth outcomes for White mothers. In fully stratified models wherein exposures and outcomes were stratified by race, negative Black sentiment was associated with adverse birth outcomes for Black mothers. Results were less consistent when examining associations between Latinx and Asian sentiment and birth outcomes for Latinx and Asian mothers, respectively, and null for White sentiment on White mothers’ birth outcomes. Together, our findings suggest that area-level sentiment toward minoritized groups is associated with negative birth outcomes for the population as a whole. Furthermore, the greatest and most consistent disparities were observed for Black mothers.

This study extends our understanding of racial health disparities. Racism is an organized system of racial hierarchy that structures risks, opportunities, and resources within a society [17]. Expressions of negativity toward minoritized groups demonstrating an ideology of racial inferiority in the values, language, imagery, symbols, and unstated assumptions of the larger society are particularly salient, working to uphold other forms of racism (eg, structural, institutional, personally mediated, and internalized). Our work suggests that negative sentiment of racial or ethnic minoritized groups explains birth outcomes among White mothers also. This aligns with research showing that policies attempting to limit the rights of a minority group have negative ramifications for the majority group also. For instance, McGhee [49] outlines how the closing of public pools across America in the 1950s—policy decisions made to deny Black people access—resulted in the elimination of this public amenity for all people, including White people. Structurally racist policies disproportionately impact people of color but also have negative consequences for all, including health outcomes for White people [49-51]. For example, racial animus toward racially minoritized groups has led to opposition to social policies and programs, such as the expansion of the Affordable Care Act [52] a policy that would also benefit a large proportion of White people. Previous research on structural racism shows that racial segregation is associated with adverse birth outcomes among racial minorities and White people [53-55], exemplifying Camara P Jones’ (former President of the American Public Health Association) [56] statement that “racism saps the strength of the whole society.”

Our findings add to the growing body of work that examines racism and health disparities for multiple racial or ethnic groups. Although the majority of the research has examined pregnancy-related health disparities for Black people, emerging research is beginning to tease apart commonalities and differences experienced by different racial groups.

Our study also advances research aiming to understand how the social and cultural environment can shape health. Social media represent a fertile space for stigmatizing language, stereotype representations, slurs, and hostile speech. As more social interactions take place on internet, it is imperative to track and monitor this space and its potential impact on health and well-being, particularly for minoritized and stigmatized racial groups. Over the last decade, we have seen a trend of increasing negative racial sentiment referencing racial and ethnic minorities. We have previously found that racialized events have led to shifts in expressed racial sentiment. For example, the killing of George Floyd was followed by a temporary decline in negative Black sentiment in 2020 [57], and negative Asian sentiment spiked during March 2020 with the emergence of the COVID-19 pandemic [58]. These events, including the resurgence of the Black Lives Matter movement in 2020, changed the sentiment and volume of discussions related to race and racism on the web [57]. These events were commonly characterized by a rapid change in racial sentiment, lasting a few weeks, followed by a return to near baseline levels [57-59]. In 2020, we saw associations between negative sentiment and adverse birth outcomes remaining comparable to other years.

The current findings also extend our understanding of the geography of racial health disparities in the United States. Our analysis included area-level racial sentiment measured at the state level. States vary in social norms, laws, and policies. However, cultural racism can also vary at lower geographic levels. There may be a bidirectional influence of tweets and state-level policies such that policies may impact tweets and tweets may in turn influence the support of new policies. We conducted analyses at the county level but considered these sensitivity analyses because approximately 40% of the Twitter data had missing county location information. Furthermore, Twitter data for less populated counties were relatively sparse, which may lead to biased estimates of racial sentiment. The geographic identifiers in the US natality files only include state and county identifiers. A valuable future direction would be to examine associations at a more granular resolution for studies looking at cultural racism and health in other data.

A major strength of this study comes from the use of social media–derived racial sentiment. Traditional approaches to measuring racial attitudes, such as survey measures or experimental approaches, are often time-consuming and expensive to conduct. These approaches provide a time-limited snapshot of sentiment, unlike the temporally rich data that can be obtained from social media posts. This temporally and geographically rich data thus makes it possible to look at changes in health disparities as a function of local health policies as well as race-related events, such as Black Lives Matter protests [57,58].

This study is not without limitations. For this paper, we used our trained SVM model for sentiment analysis, as we have used this model in previous publications [57,58], including our paper examining racial sentiment and birth outcomes for 2015-2017 [34]. Changing the sentiment analysis model may change the findings. We have found a high level of accuracy and a high F1 score with this model. To be most comparable to our other papers, we used this sentiment model here. The keyword list is not exhaustive, but we attempted to be as comprehensive as possible by using words used by the US Census, a racial slurs database, and previous studies, while also balancing search constraints that limited the number of characters that can be used for searches to 1024 characters. Data collected is what people were willing to express on Twitter, where negative content tends to spread faster [60] and, therefore, may be overestimated. Furthermore, we analyzed tweets referencing different racial groups for sentiment or emotional tone, which is different from previous work that has more directly focused on hate speech and racial slurs, for example [61-63]. However, we believe this approach to be sensitive to subtle expressions of racial attitudes that are more representative of the area-level culture and have previously found that such measures are associated with explicit and implicit forms of racial bias [64]. Other limitations include our exclusive focus on English-language tweets. Future research can examine non-English language tweets referencing minoritized populations to evaluate how the racial sentiment of English and non-English tweets may differ. Approximately 1 in 5 US adults use Twitter [31]. Racial or ethnic minoritized populations are slightly overrepresented on Twitter compared to the US general population. For example, 17% of adult Twitter users are Latinx compared to 15% of the US adult population [65]. Twitter users skew younger than the general US population, with 42% of Twitter users being between the ages of 18 and 29 years [66], whereas those aged 19-34 years account for only 20.8% of the US population [67]. However, Twitter users have become more similar to the US population in terms of education over time. In 2016, Twitter users tended to be more educated than the US population [68]. In 2021, 33% of adult Twitter users have a college or graduate degree [66] compared to 38% of US adults 25 years and older with a college or graduate degree [69]. Visitors tweeting from a location may express different sentiments compared to residents of places. Twitter users differ in their frequency of tweeting with the majority of Twitter users being less frequent users. However, in our data on race-related tweets, tweets from users tweeting more than 1000 times per year represent less than 1% (n=1066) of the data.

Despite these limitations, the Twitter data provide a broad signal for the social context and the online environment people may be exposed to. The focus is not on individual users at one particular time point or place but rather on the patterns of associations with birth outcomes in the United States by using a continuous random sample of US tweets from 2011 to 2021.

The state-level aggregation of racial sentiment also makes it challenging to tease apart the contribution of state politics (structural) and sentiment (cultural) to birth outcomes. For instance, Medicaid is known to mitigate racial health disparities [70], but states vary in the degree to which they expanded Medicaid benefits to their constituents [71]. Sentiment toward minority race groups is an important, yet hard-to-measure factor that may contribute to the instantiation of policies intended to impact these groups [17]. Given that cultural and structural racism are mutually reinforcing factors, areas that are more welcoming to racial or ethnic minorities may have greater safety net programs for example. Future work can build on the current findings to further examine the mutually reinforcing nature of state-level sentiment and policies contributing to racial health disparities.

Policies that support racial literacy and cultural competency training as well as policies and supports that promote racial equity more broadly (housing, education, criminal justice, media, and city planning) and establish an inclusive social environment accepting of all races and cultures may improve population health, decreasing the overall risk of adverse birth outcomes and reducing racial-birth outcome disparities. Additionally, our results suggest that policies and practices that ostracize, stigmatize, or otherwise isolate certain racial or ethnic groups and increase hostility directed toward them may exacerbate adverse birth outcomes, which would perpetuate the impact of racism or discrimination on future generations.

### Conclusions

This study represents advancement in our ability to assess racial sentiment at a more fine-grained level as a means to provide more detailed explanations for racial disparities in birth outcomes. Social media provide a unique opportunity to examine racial sentiment across time, geographic location, and for different racial groups. By examining the relationship between sentiment and birth outcomes across racial groups, we are better able to assess the specificity and generalizability of cultural racism in the outcomes for different groups. Future work can build on our findings, and use this work to inform policy aimed at reducing racial disparities in health.

## Appendix

Multimedia Appendix 1 Supplementary figures, tables, and code.

## Abbreviations

FIPS: Federal Information Processing Standards
LBW: low birth weight
PTB: preterm birth
SVM: support vector machine

## Data Availability

Twitter data were collected using Twitter’s Application Programming Interface for Academic Research. More information on applying for access can be found at [72].

The paper also uses restricted US natality files with geographic identifiers. The files were obtained after submitting a research proposal to and obtaining approval for data access from the National Center for Health Statistics. More information on applying can be found at [73]. We have added example code to run the analyses in Multimedia Appendix 1.
