# Supplementary material for: Examining Twitter-Derived Negative Racial Sentiment as Indicators of Cultural Racism: Observational Associations With Preterm Birth and Low Birth Weight Among a Multiracial Sample of Mothers, 2011-2021
Source: J Med Internet Res. 2023 Apr 28;25:e44990. doi: 10.2196/44990 (PMC10182466; doi:10.2196/44990)
Supplement: Multimedia Appendix 1 [file jmir_v25i1e44990_app1.docx]

Online Supplementary Figures, Tables, and Code


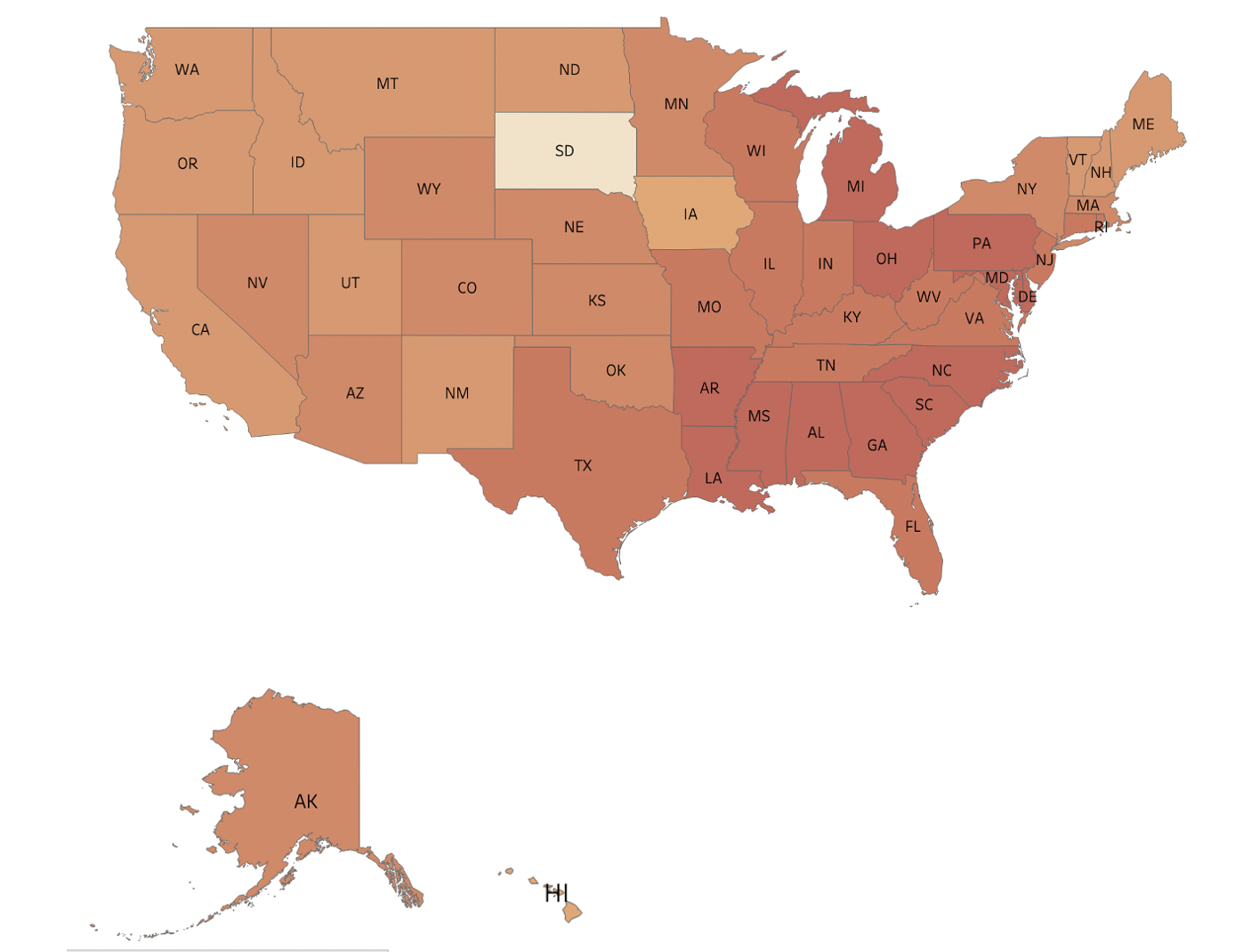


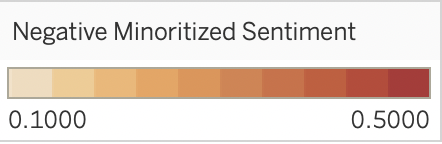


**Figure S1.** Geographic distribution of averaged negative sentiment of tweets referencing minoritized groups for 2011-2021.


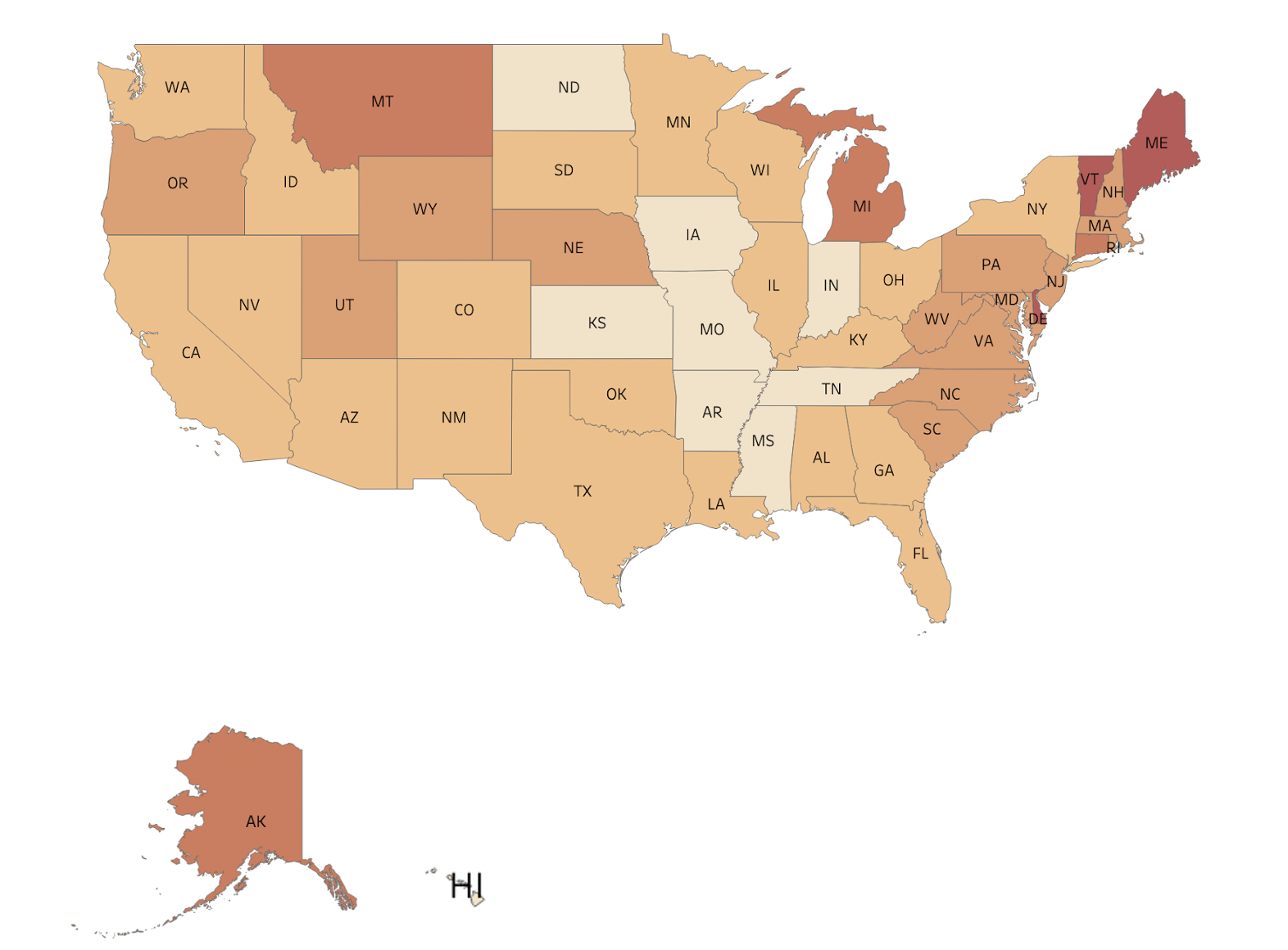


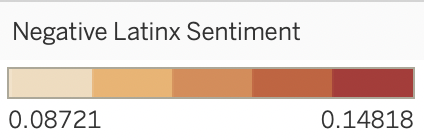


**Figure S2.** Geographic distribution of averaged negative sentiment of tweets referencing Latinx individuals for 2011-2021.


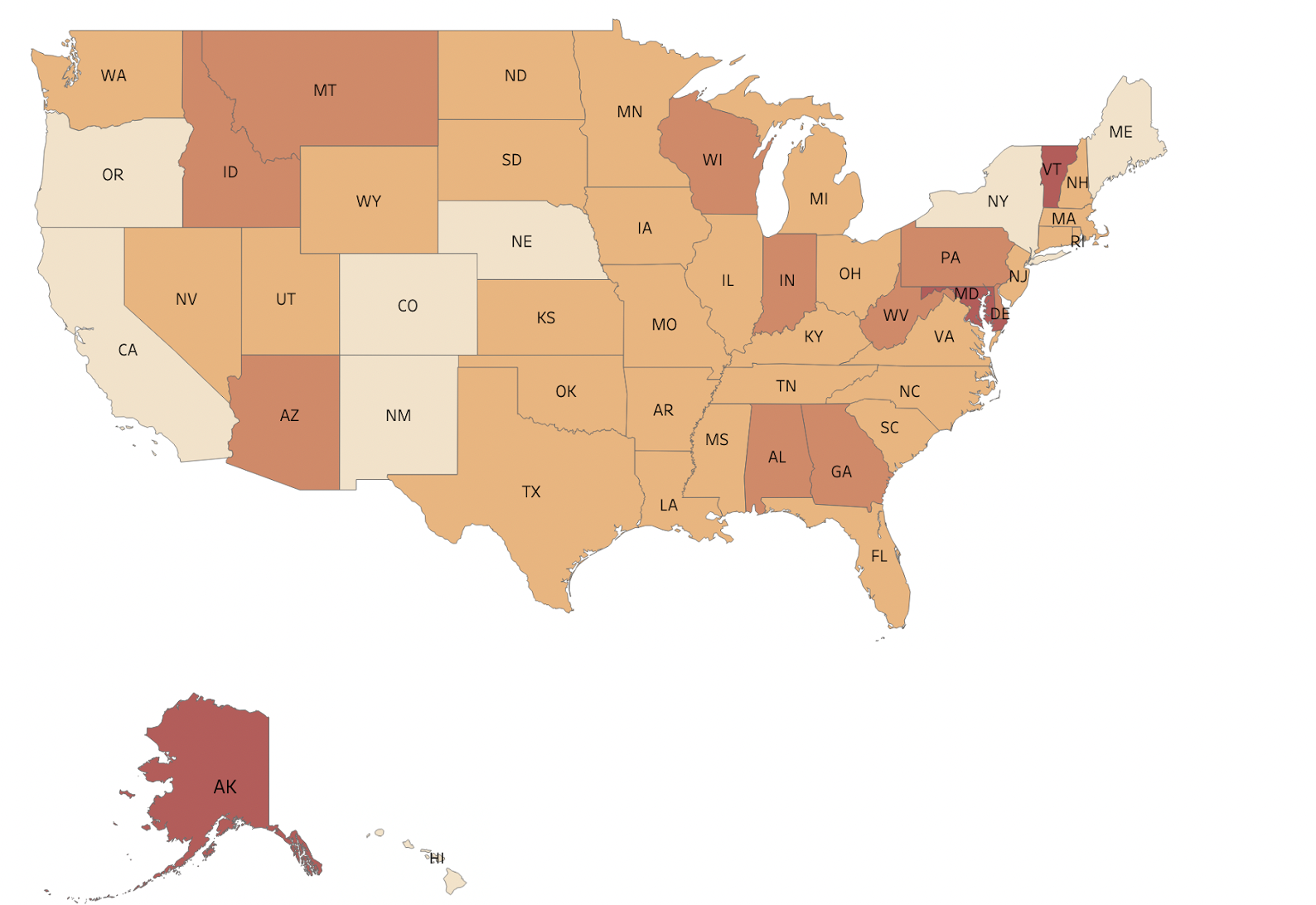


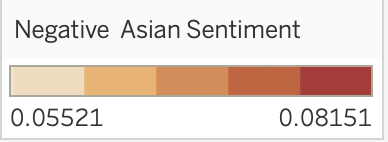


**Figure S3.** Geographic distribution of averaged negative sentiment of tweets referencing Asian individuals for 2011-2021

| **Table S1.** Race and ethnicity related keywords used in the Twitter data collection   \| **Items** \| **Race** \| \| --- \| --- \| \| asian \| asian \| \| asians \| asian \| \| filipino \| asian \| \| japanese \| asian \| \| korean \| asian \| \| nepal \| asian \| \| pacific islander \| asian \| \| thai \| asian \| \| vietnamese \| asian \| \| chinese \| asian \| \| #blacklivesmatter \| black \| \| black boy \| black \| \| black boys \| black \| \| black girl \| black \| \| black man \| black \| \| black men \| black \| \| black people \| black \| \| black ppl \| black \| \| black woman \| black \| \| black women \| black \| \| blackgirls \| black \| \| blacks \| black \| \| blm \| black \| \| negro \| black \| \| nigga \| black \| \| niggas \| black \| \| nigger \| black \| \| slavery \| black \| \| border security \| hispanic \| \| border wall \| hispanic \| \| cuban \| hispanic \| \| domican \| hispanic \| \| hispanic \| hispanic \| \| latina \| hispanic \| \| latino \| hispanic \| \| latinos \| hispanic \| \| latinx \| hispanic \| \| mexican \| hispanic \| \| mexicans \| hispanic \| \| puerto rican \| hispanic \| \| illegal aliens \| immigrant \| \| illegal immigrants \| immigrant \| \| illegals \| immigrant \| \| immigrant \| immigrant \| \| immigrants \| immigrant \| \| immigration \| immigrant \| \| migrant \| immigrant \| \| migrants \| immigrant \| \| sanctuary \| immigrant \| \| undocumented \| immigrant \| \| jewish \| jewish \| \| jews \| jewish \| \| afghan \| middle eastern \| \| afghanistan \| middle eastern \| \| islam \| middle eastern \| \| muslim \| middle eastern \| \| muslims \| middle eastern \| \| taliban \| middle eastern \| \| yemeni \| middle eastern \| \| minorities \| minority \| \| people of color \| minority \| \| poc \| minority \| \| racism \| minority \| \| racist \| minority \| \| racists \| minority \| \| biracial \| multi-race \| \| interracial \| multi-race \| \| cherokee \| native american \| \| native american \| native american \| \| native americans \| native american \| \| sioux \| native american \| \| refugee \| refugee \| \| caucasian \| white \| \| kkk \| white \| \| nazis \| white \| \| proud boys \| white \| \| redneck \| white \| \| white boy \| white \| \| white boys \| white \| \| white girl \| white \| \| white girls \| white \| \| white guy \| white \| \| white man \| white \| \| white men \| white \| \| white people \| white \| \| white ppl \| white \| \| white woman \| white \| \| white women \| white \| \| whites \| white \|  \| **Table S2.** Top twenty terms by year \| \| --- \|  \|  \| 2011 \| \| 2012 \| \| 2013 \| \| \| --- \| --- \| --- \| --- \| --- \| --- \| --- \| \|  \| Term \| N \| Term \| N \| Term \| N \| \|  \| nigga \| 1,083,196 \| nigga \| 2,510,762 \| nigga \| 3,543,479 \| \|  \| niggas \| 479,348 \| niggas \| 1,145,977 \| niggas \| 1,772,567 \| \|  \| mexican \| 74,139 \| mexican \| 169,015 \| mexican \| 231,165 \| \|  \| chinese \| 61,489 \| chinese \| 134,433 \| chinese \| 178,316 \| \|  \| asian \| 44,158 \| asian \| 94,390 \| asian \| 126,874 \| \|  \| japanese \| 34,070 \| racist \| 53,382 \| white girl \| 89,794 \| \|  \| thai \| 23,113 \| japanese \| 49,406 \| racist \| 69,042 \| \|  \| racist \| 21,604 \| black people \| 45,961 \| white girls \| 59,020 \| \|  \| black people \| 19,218 \| white girl \| 42,849 \| japanese \| 58,263 \| \|  \| white girl \| 18,199 \| thai \| 38,323 \| black people \| 56,801 \| \|  \| white people \| 15,177 \| white people \| 34,580 \| white people \| 55,852 \| \|  \| jewish \| 13,869 \| white girls \| 26,485 \| sioux \| 49,779 \| \|  \| korean \| 12,965 \| korean \| 24,682 \| thai \| 47,227 \| \|  \| white boy \| 11,507 \| white boy \| 24,083 \| redneck \| 34,514 \| \|  \| negro \| 10,327 \| jewish \| 23,534 \| mexicans \| 33,562 \| \|  \| white girls \| 9,734 \| mexicans \| 22,391 \| white boy \| 32,556 \| \|  \| afghanistan \| 9,259 \| negro \| 19,161 \| korean \| 30,063 \| \|  \| cuban \| 9,125 \| redneck \| 18,895 \| jewish \| 29,005 \| \|  \| mexicans \| 8,949 \| sioux \| 18,591 \| hispanic \| 25,639 \| \|  \| sioux \| 8,422 \| asians \| 18,144 \| asians \| 25,636 \|  \| 2014 \| \| 2015 \| \| 2016 \| \| \| --- \| --- \| --- \| --- \| --- \| --- \| \| Term \| N \| Term \| N \| Term \| N \| \| nigga \| 4,239,142 \| nigga \| 3,732,904 \| nigga \| 2,099,051 \| \| niggas \| 2,172,739 \| niggas \| 2,084,926 \| niggas \| 1,256,616 \| \| mexican \| 267,672 \| mexican \| 254,051 \| racist \| 260,870 \| \| chinese \| 195,106 \| chinese \| 205,177 \| mexican \| 177,033 \| \| asian \| 140,708 \| racist \| 178,178 \| chinese \| 139,435 \| \| white girl \| 124,401 \| asian \| 132,154 \| racism \| 112,951 \| \| racist \| 108,251 \| white people \| 127,267 \| white people \| 104,156 \| \| white people \| 97,270 \| racism \| 97,968 \| asian \| 83,202 \| \| white girls \| 88,633 \| japanese \| 94,929 \| muslim \| 79,365 \| \| black people \| 78,726 \| #blacklivesmatter \| 90,090 \| black people \| 75,537 \| \| japanese \| 73,985 \| black people \| 89,984 \| #blacklivesmatter \| 70,342 \| \| thai \| 57,925 \| muslim \| 82,802 \| blacks \| 69,934 \| \| sioux \| 47,814 \| white girl \| 80,354 \| japanese \| 63,799 \| \| racism \| 46,900 \| thai \| 64,171 \| muslims \| 54,139 \| \| white boy \| 40,683 \| blacks \| 59,793 \| immigration \| 49,071 \| \| jewish \| 38,344 \| muslims \| 54,849 \| islam \| 43,385 \| \| mexicans \| 37,545 \| jewish \| 53,469 \| thai \| 42,959 \| \| korean \| 37,286 \| white girls \| 52,587 \| cuban \| 41,607 \| \| immigration \| 32,969 \| islam \| 50,636 \| hispanic \| 40,805 \| \| cuban \| 32,580 \| immigration \| 48,420 \| white girl \| 40,678 \|  \| 2017 \| \| 2018 \| \| 2019 \| \| \| --- \| --- \| --- \| --- \| --- \| --- \| \| Term \| N \| Term \| N \| Term \| N \| \| nigga \| 1,164,782 \| nigga \| 1,218,724 \| nigga \| 1,084,060 \| \| niggas \| 802,328 \| niggas \| 823,882 \| niggas \| 758,817 \| \| racist \| 215,080 \| racist \| 339,668 \| racist \| 328,467 \| \| mexican \| 115,241 \| racism \| 130,035 \| racism \| 124,476 \| \| chinese \| 97,246 \| mexican \| 126,392 \| mexican \| 106,858 \| \| racism \| 95,702 \| immigration \| 99,241 \| chinese \| 84,238 \| \| white people \| 75,698 \| chinese \| 93,362 \| immigration \| 72,954 \| \| muslim \| 62,991 \| white people \| 84,821 \| white people \| 71,261 \| \| asian \| 54,573 \| immigrants \| 77,895 \| black people \| 68,583 \| \| immigration \| 52,186 \| black people \| 75,325 \| immigrants \| 58,229 \| \| black people \| 51,828 \| illegals \| 64,979 \| illegals \| 54,788 \| \| japanese \| 50,727 \| asian \| 61,120 \| asian \| 54,240 \| \| nazis \| 50,398 \| japanese \| 56,079 \| jewish \| 53,458 \| \| immigrants \| 45,493 \| immigrant \| 55,978 \| japanese \| 50,240 \| \| jewish \| 40,544 \| jewish \| 50,142 \| muslim \| 44,462 \| \| korean \| 36,693 \| muslim \| 46,162 \| slavery \| 38,904 \| \| muslims \| 35,686 \| korean \| 44,557 \| jews \| 37,041 \| \| blacks \| 32,111 \| blacks \| 39,938 \| black women \| 36,625 \| \| slavery \| 32,070 \| slavery \| 39,760 \| immigrant \| 34,082 \| \| islam \| 31,255 \| racists \| 34,594 \| blacks \| 32,745 \|  \| 2020 \| \| 2021 \| \| \| --- \| --- \| --- \| --- \| \| Term \| N \| Term \| N \| \| nigga \| 920,679 \| nigga \| 445,852 \| \| niggas \| 673,737 \| niggas \| 332,284 \| \| racist \| 393,678 \| racist \| 161,778 \| \| racism \| 198,207 \| racism \| 81,953 \| \| #blacklivesmatter \| 171,895 \| afghanistan \| 60,291 \| \| blm \| 147,512 \| blm \| 52,911 \| \| black people \| 133,680 \| mexican \| 51,835 \| \| chinese \| 117,142 \| black people \| 50,817 \| \| white people \| 84,854 \| chinese \| 49,024 \| \| mexican \| 76,584 \| asian \| 45,644 \| \| asian \| 55,353 \| white people \| 37,709 \| \| black man \| 53,868 \| taliban \| 29,200 \| \| blacks \| 51,017 \| jewish \| 27,936 \| \| black women \| 50,775 \| japanese \| 26,415 \| \| black men \| 45,571 \| slavery \| 24,444 \| \| slavery \| 42,244 \| black women \| 23,719 \| \| racists \| 42,242 \| #blacklivesmatter \| 20,968 \| \| jewish \| 36,630 \| black man \| 20,922 \| \| japanese \| 36,496 \| immigrants \| 18,118 \| \| black woman \| 32,902 \| blacks \| 17,716 \|   **Table S3.** Associations between state-level racial sentiment towards minoritized and low birth weight for full sample, minoritized mothers, and White mothers by year | | | | |
| --- | --- | --- | --- | --- | --- | --- | --- | --- | --- | --- | --- | --- | --- | --- | --- | --- | --- | --- | --- | --- | --- | --- | --- | --- | --- | --- | --- | --- | --- | --- | --- | --- | --- | --- | --- | --- | --- | --- | --- | --- | --- | --- | --- | --- | --- | --- | --- | --- | --- | --- | --- | --- | --- | --- | --- | --- | --- | --- | --- | --- | --- | --- | --- | --- | --- | --- | --- | --- | --- | --- | --- | --- | --- | --- | --- | --- | --- | --- | --- | --- | --- | --- | --- | --- | --- | --- | --- | --- | --- | --- | --- | --- | --- | --- | --- | --- | --- | --- | --- | --- | --- | --- | --- | --- | --- | --- | --- | --- | --- | --- | --- | --- | --- | --- | --- | --- | --- | --- | --- | --- | --- | --- | --- | --- | --- | --- | --- | --- | --- | --- | --- | --- | --- | --- | --- | --- | --- | --- | --- | --- | --- | --- | --- | --- | --- | --- | --- | --- | --- | --- | --- | --- | --- | --- | --- | --- | --- | --- | --- | --- | --- | --- | --- | --- | --- | --- | --- | --- | --- | --- | --- | --- | --- | --- | --- | --- | --- | --- | --- | --- | --- | --- | --- | --- | --- | --- | --- | --- | --- | --- | --- | --- | --- | --- | --- | --- | --- | --- | --- | --- | --- | --- | --- | --- | --- | --- | --- | --- | --- | --- | --- | --- | --- | --- | --- | --- | --- | --- | --- | --- | --- | --- | --- | --- | --- | --- | --- | --- | --- | --- | --- | --- | --- | --- | --- | --- | --- | --- | --- | --- | --- | --- | --- | --- | --- | --- | --- | --- | --- | --- | --- | --- | --- | --- | --- | --- | --- | --- | --- | --- | --- | --- | --- | --- | --- | --- | --- | --- | --- | --- | --- | --- | --- | --- | --- | --- | --- | --- | --- | --- | --- | --- | --- | --- | --- | --- | --- | --- | --- | --- | --- | --- | --- | --- | --- | --- | --- | --- | --- | --- | --- | --- | --- | --- | --- | --- | --- | --- | --- | --- | --- | --- | --- | --- | --- | --- | --- | --- | --- | --- | --- | --- | --- | --- | --- | --- | --- | --- | --- | --- | --- | --- | --- | --- | --- | --- | --- | --- | --- | --- | --- | --- | --- | --- | --- | --- | --- | --- | --- | --- | --- | --- | --- | --- | --- | --- | --- | --- | --- | --- | --- | --- | --- | --- | --- | --- | --- | --- | --- | --- | --- | --- | --- | --- | --- | --- | --- | --- | --- | --- | --- | --- | --- | --- | --- | --- | --- | --- | --- | --- | --- | --- | --- | --- | --- | --- | --- | --- | --- | --- | --- | --- | --- | --- | --- | --- | --- | --- | --- | --- | --- | --- | --- | --- | --- | --- | --- | --- | --- | --- | --- | --- | --- | --- | --- | --- | --- | --- | --- | --- | --- | --- | --- | --- | --- | --- | --- | --- | --- | --- | --- | --- | --- | --- | --- | --- | --- | --- | --- | --- | --- | --- | --- | --- | --- | --- | --- | --- | --- | --- | --- | --- | --- | --- | --- | --- | --- | --- | --- | --- | --- | --- | --- | --- | --- | --- | --- | --- | --- | --- | --- | --- | --- | --- | --- | --- | --- | --- | --- | --- | --- | --- | --- | --- | --- | --- | --- | --- | --- | --- | --- | --- | --- | --- | --- | --- | --- | --- | --- | --- | --- | --- | --- | --- | --- | --- | --- | --- | --- | --- | --- | --- | --- | --- | --- | --- | --- | --- | --- | --- | --- | --- | --- | --- | --- | --- | --- | --- | --- | --- | --- | --- | --- | --- | --- | --- | --- | --- | --- | --- | --- | --- | --- | --- | --- | --- | --- | --- | --- | --- | --- | --- | --- | --- | --- | --- | --- | --- | --- | --- | --- | --- | --- | --- | --- | --- | --- | --- | --- | --- | --- | --- | --- | --- | --- | --- | --- | --- | --- | --- | --- | --- | --- | --- | --- | --- | --- | --- | --- | --- | --- | --- | --- | --- | --- | --- | --- | --- | --- | --- | --- | --- | --- | --- | --- | --- | --- | --- | --- | --- | --- | --- | --- | --- | --- | --- | --- | --- | --- | --- | --- | --- | --- | --- | --- | --- | --- | --- | --- | --- | --- | --- | --- | --- | --- | --- | --- | --- | --- | --- | --- | --- | --- | --- | --- | --- | --- | --- | --- | --- | --- | --- | --- | --- | --- | --- | --- | --- | --- | --- | --- | --- | --- | --- | --- | --- | --- | --- | --- | --- | --- | --- | --- | --- | --- | --- | --- | --- | --- | --- | --- |
| **Year** | **Data** | **Total** | **Minoritized Groups** | **Whites** |
| **2011** | 2nd tertile vs 1^st^ (lowest) | 1.09 (0.98-1.21) | 1.15 (1.05-1.25) | 1.04 (0.91-1.19) |
|  | 3rd tertile vs 1^st^ (lowest) | 1.15 (1.02-1.3) | 1.18 (1.05-1.33) | 1.11 (0.96-1.28) |
|  | Number | 3,066,252 | 1,485,256 | 1,679,831 |
| **2012** | 2nd tertile vs 1^st^ (lowest) | 1.08 (0.97-1.19) | 1.11 (1.01-1.23) | 1.04 (0.92-1.18) |
|  | 3rd tertile vs 1^st^ (lowest) | 1.14 (1.01-1.28) | 1.16 (1.02-1.31) | 1.11 (0.98-1.26) |
|  | Number | 3,158,279 | 1,533,938 | 1,728,374 |
| **2013** | 2nd tertile vs 1^st^ (lowest) | 1.13 (1.06-1.21) | 1.18 (1.11-1.24) | 1.11 (1.01-1.22) |
|  | 3rd tertile vs 1^st^ (lowest) | 1.13 (1.04-1.23) | 1.20 (1.11-1.29) | 1.10 (0.98-1.23) |
|  | Number | 3,210,323 | 1,553,321 | 1,764,473 |
| **2014** | 2nd tertile vs 1^st^ (lowest) | 1.14 (1.07-1.21) | 1.15 (1.09-1.22) | 1.15 (1.06-1.25) |
|  | 3rd tertile vs 1^st^ (lowest) | 1.15 (1.07-1.23) | 1.17 (1.09-1.26) | 1.15 (1.05-1.25) |
|  | Number | 3,454,046 | 1,703,803 | 1,859,793 |
| **2015** | 2nd tertile vs 1^st^ (lowest) | 1.05 (0.96-1.15) | 1.08 (1.0-1.18) | 1.02 (0.91-1.14) |
|  | 3rd tertile vs 1^st^ (lowest) | 1.11 (1.01-1.21) | 1.13 (1.03-1.25) | 1.09 (0.99-1.2) |
|  | Number | 3,553,772 | 1,776,360 | 1,895,620 |
| **2016** | 2nd tertile vs 1^st^ (lowest) | 1.11 (1.04-1.19) | 1.13 (1.05-1.21) | 1.11 (1.02-1.21) |
|  | 3rd tertile vs 1^st^ (lowest) | 1.13 (1.06-1.19) | 1.11 (1.03-1.2) | 1.14 (1.06-1.22) |
|  | Number | 3,603,791 | 1,823,325 | 1,904,038 |
| **2017** | 2nd tertile vs 1^st^ (lowest) | 1.06 (0.99-1.13) | 1.04 (0.98-1.1) | 1.08 (1.0-1.17) |
|  | 3rd tertile vs 1^st^ (lowest) | 1.08 (1.03-1.13) | 1.07 (1.02-1.12) | 1.10 (1.04-1.17) |
|  | Number | 3,115,637 | 1,486,487 | 1,734,962 |
| **2018** | 2nd tertile vs 1^st^ (lowest) | 1.07 (1.0-1.13) | 1.04 (0.98-1.1) | 1.09 (1.01-1.18) |
|  | 3rd tertile vs 1^st^ (lowest) | 1.05 (0.99-1.11) | 1.03 (0.97-1.09) | 1.06 (0.99-1.14) |
|  | Number | 3,073,239 | 1,469,225 | 1,707,401 |
| **2019** | 2nd tertile vs 1^st^ (lowest) | 0.98 (0.9-1.07) | 0.97 (0.9-1.05) | 1.00 (0.9-1.11) |
|  | 3rd tertile vs 1^st^ (lowest) | 1.03 (0.96-1.11) | 1.03 (0.95-1.11) | 1.04 (0.95-1.14) |
|  | Number | 3,054,213 | 1,475,115 | 1,679,716 |
| **2020** | 2nd tertile vs 1^st^ (lowest) | 1.07 (1.0-1.14) | 1.05 (0.98-1.12) | 1.09 (1.0-1.18) |
|  | 3rd tertile vs 1^st^ (lowest) | 1.07 (1.0-1.14) | 1.05 (0.98-1.13) | 1.08 (1.0-1.17) |
|  | Number | 2,968,973 | 1,440,734 | 1,623,019 |
| **2021** | 2nd tertile vs 1^st^ (lowest) | 1.03 (0.94-1.13) | 1.04 (0.95-1.14) | 1.04 (0.93-1.15) |
|  | 3rd tertile vs 1^st^ (lowest) | 1.04 (0.95-1.14) | 1.05 (0.96-1.14) | 1.05 (0.95-1.16) |
|  | Number | 3,008,652 | 1,443,175 | 1,659,468 |

| **Table S4.** Associations between state-level racial sentiment towards minoritized and preterm birth for full sample, minoritized mothers, and White mothers by year | | | | |
| --- | --- | --- | --- | --- |
| **Year** | **Data** | **Total** | **Minoritized Groups** | **Whites** |
| **2011** | 2nd tertile vs 1^st^ (lowest) | 1.10 (1.03-1.17) | 1.13 (1.07-1.19) | 1.08 (0.99-1.17) |
|  | 3rd tertile vs 1^st^ (lowest) | 1.14 (1.05-1.25) | 1.16 (1.07-1.25) | 1.13 (1.01-1.26) |
|  | Number | 3,068,089 | 1,486,155 | 1,680,812 |
| **2012** | 2nd tertile vs 1^st^ (lowest) | 1.07 (1.0-1.14) | 1.08 (1.02-1.15) | 1.07 (0.99-1.15) |
|  | 3rd tertile vs 1^st^ (lowest) | 1.10 (1.01-1.21) | 1.11 (1.01-1.21) | 1.1 (0.99-1.22) |
|  | Number | 3,160,473 | 1,535,048 | 1,729,525 |
| **2013** | 2nd tertile vs 1^st^ (lowest) | 1.08 (1.02-1.14) | 1.11 (1.05-1.18) | 1.06 (0.99-1.13) |
|  | 3rd tertile vs 1^st^ (lowest) | 1.08 (0.99-1.18) | 1.12 (1.03-1.22) | 1.07 (0.97-1.18) |
|  | Number | 3,212,603 | 1,554,493 | 1,765,632 |
| **2014** | 2nd tertile vs 1^st^ (lowest) | 1.07 (1.01-1.14) | 1.09 (1.03-1.16) | 1.07 (1.0-1.15) |
|  | 3rd tertile vs 1^st^ (lowest) | 1.09 (1.01-1.17) | 1.10 (1.02-1.19) | 1.09 (0.99-1.19) |
|  | Number | 3,455,704 | 1,704,581 | 1,860,703 |
| **2015** | 2nd tertile vs 1^st^ (lowest) | 1.05 (1.0-1.11) | 1.06 (1.01-1.12) | 1.06 (0.99-1.13) |
|  | 3rd tertile vs 1^st^ (lowest) | 1.10 (1.03-1.17) | 1.09 (1.02-1.18) | 1.11 (1.03-1.2) |
|  | Number | 3,555,905 | 1,777,295 | 1,896,892 |
| **2016** | 2nd tertile vs 1^st^ (lowest) | 1.07 (1.02-1.12) | 1.08 (1.03-1.13) | 1.07 (1.01-1.14) |
|  | 3rd tertile vs 1^st^ (lowest) | 1.08 (1.02-1.15) | 1.09 (1.02-1.15) | 1.09 (1.01-1.17) |
|  | Number | 3,606,701 | 1,824,683 | 1,905,676 |
| **2017** | 2nd tertile vs 1^st^ (lowest) | 1.03 (0.97-1.09) | 1.02 (0.97-1.07) | 1.04 (0.97-1.13) |
|  | 3rd tertile vs 1^st^ (lowest) | 1.06 (1.0-1.13) | 1.07 (1.01-1.13) | 1.06 (0.99-1.15) |
|  | Number | 3,117,193 | 1,487,282 | 1,735,764 |
| **2018** | 2nd tertile vs 1^st^ (lowest) | 1.08 (1.01-1.15) | 1.07 (1.01-1.14) | 1.09 (1.0-1.19) |
|  | 3rd tertile vs 1^st^ (lowest) | 1.02 (0.95-1.1) | 1.01 (0.94-1.08) | 1.04 (0.95-1.14) |
|  | Number | 3,074,726 | 1,469,910 | 1,708,235 |
| **2019** | 2nd tertile vs 1^st^ (lowest) | 1.06 (1.00-1.13) | 1.05 (0.99-1.10) | 1.08 (0.99-1.17) |
|  | 3rd tertile vs 1^st^ (lowest) | 1.01 (0.94-1.09) | 0.99 (0.92-1.07) | 1.03 (0.94-1.13) |
|  | Number | 3,056,152 | 1,476,078 | 1,680,757 |
| **2020** | 2nd tertile vs 1^st^ (lowest) | 1.07 (1.02-1.13) | 1.07 (1.03-1.12) | 1.06 (0.98-1.16) |
|  | 3rd tertile vs 1^st^ (lowest) | 1.00 (0.94-1.06) | 1.00 (0.95-1.05) | 1.00 (0.91-1.09) |
|  | Number | 2,970,441 | 1,441,493 | 1,623,755 |
| **2021** | 2nd tertile vs 1^st^ (lowest) | 0.99 (0.92-1.07) | 1.00 (0.92-1.07) | 1.00 (0.91-1.09) |
|  | 3rd tertile vs 1^st^ (lowest) | 1.02 (0.94-1.1) | 1.03 (0.95-1.11) | 1.02 (0.92-1.14) |
|  | Number | 3,010,027 | 1,443,889 | 1,660,159 |

| **Table S5.** Race and ethnic associations between state-level negative sentiment and low birth weight by year | | | | | |
| --- | --- | --- | --- | --- | --- |
| **Year** | **Data** | **Negative Black sentiment, birth outcomes among Black mothers** | **Negative Asian sentiment, birth outcomes among Asian mothers** | **Negative Latinx sentiment, birth outcomes among Latinx mothers** | **Negative White sentiment, birth outcomes among White mothers** |
| 2011 | 2nd tertile vs 1^st^ (lowest) | 1.07 (0.97-1.17) | 1.09 (1.01-1.19) | 0.89 (0.76-1.04) | 1.04 (0.95-1.14) |
|  | 3rd tertile vs 1^st^ (lowest) | 1.02 (0.91-1.14) | 1.00 (0.93-1.08) | 0.96 (0.82-1.13) | 1.07 (0.99-1.17) |
|  | Number | 422,831 | 207,617 | 755,973 | 1,679,831 |
| 2012 | 2nd tertile vs 1^st^ (lowest) | 1.13 (1.02-1.24) | 1.04 (0.97-1.12) | 1.05 (0.92-1.2) | 1.08 (0.98-1.19) |
|  | 3rd tertile vs 1^st^ (lowest) | 1.08 (0.99-1.18) | 1.05 (0.95-1.16) | 1.09 (0.96-1.22) | 1.11 (1.02-1.22) |
|  | Number | 441,934 | 229,249 | 758,722 | 1,728,374 |
| 2013 | 2nd tertile vs 1^st^ (lowest) | 1.10 (0.99-1.22) | 1.08 (0.99-1.19) | 1.15 (1.03-1.28) | 0.94 (0.87-1.01) |
|  | 3rd tertile vs 1^st^ (lowest) | 1.06 (0.96-1.17) | 0.98 (0.87-1.1) | 1.12 (0.99-1.28) | 1.02 (0.91-1.14) |
|  | Number | 460,996 | 230,918 | 753,936 | 1,764,473 |
| 2014 | 2nd tertile vs 1^st^ (lowest) | 1.09 (1.02-1.16) | 0.99 (0.94-1.05) | 1.02 (0.94-1.12) | 1.01 (0.91-1.1) |
|  | 3rd tertile vs 1^st^ (lowest) | 1.11 (1.04-1.18) | 1.12 (1.04-1.2) | 1.16 (1.04-1.29) | 1.01 (0.91-1.13) |
|  | Number | 470,911 | 211,493 | 805,533 | 1,859,793 |
| 2015 | 2nd tertile vs 1^st^ (lowest) | 1.15 (1.11-1.2) | 0.95 (0.87-1.03) | 1.02 (0.86-1.19) | 0.95 (0.86-1.06) |
|  | 3rd tertile vs 1^st^ (lowest) | 1.15 (1.1-1.21) | 1.05 (0.95-1.17) | 1.12 (0.97-1.3) | 0.96 (0.86-1.08) |
|  | Number | 492,924 | 218,586 | 836,972 | 1,895,620 |
| 2016 | 2nd tertile vs 1^st^ (lowest) | 1.13 (1.05-1.23) | 0.94 (0.87-1.02) | 0.91 (0.84-0.99) | 0.99 (0.9-1.09) |
|  | 3rd tertile vs 1^st^ (lowest) | 1.13 (1.04-1.23) | 0.98 (0.9-1.08) | 1.06 (0.92-1.21) | 0.97 (0.9-1.04) |
|  | Number | 499,041 | 234,925 | 853,398 | 1,904,038 |
| 2017 | 2nd tertile vs 1^st^ (lowest) | 1.06 (1.01-1.11) | 0.95 (0.89-1.01) | 0.95 (0.88-1.02) | 0.95 (0.84-1.06) |
|  | 3rd tertile vs 1^st^ (lowest) | 1.06 (1.02-1.11) | 0.97 (0.86-1.09) | 1.03 (0.95-1.11) | 0.95 (0.83-1.09) |
|  | Number | 485,003 | 164,971 | 631,655 | 1,734,962 |
| 2018 | 2nd tertile vs 1^st^ (lowest) | 1.06 (1.02-1.1) | 1.08 (0.99-1.17) | 1.07 (0.96-1.19) | 1.00 (0.94-1.07) |
|  | 3rd tertile vs 1^st^ (lowest) | 1.07 (1.03-1.1) | 1.07 (1.01-1.13) | 1.06 (0.96-1.17) | 0.99 (0.93-1.05) |
|  | Number | 478,359 | 160,586 | 627,213 | 1,707,401 |
| 2019 | 2nd tertile vs 1^st^ (lowest) | 1.02 (0.96-1.09) | 0.98 (0.9-1.07) | 0.96 (0.86-1.07) | 1.01 (0.95-1.08) |
|  | 3rd tertile vs 1^st^ (lowest) | 1.02 (0.96-1.09) | 1.01 (0.93-1.1) | 1.03 (0.94-1.13) | 1.03 (0.96-1.1) |
|  | Number | 478,504 | 159,779 | 635,490 | 1,679,716 |
| 2020 | 2nd tertile vs 1^st^ (lowest) | 1.08 (1.02-1.14) | 1.04 (0.96-1.13) | 1.03 (0.92-1.14) | 1.02 (0.97-1.08) |
|  | 3rd tertile vs 1^st^ (lowest) | 1.06 (1.0-1.13) | 1.03 (0.95-1.12) | 1.01 (0.9-1.12) | 1.00 (0.93-1.07) |
|  | Number | 466,550 | 150,887 | 628,887 | 1,623,019 |
| 2021 | 2nd tertile vs 1^st^ (lowest) | 1.06 (0.98-1.16) | 0.84 (0.76-0.93) | 1.02 (0.96-1.1) | 0.98 (0.93-1.03) |
|  | 3rd tertile vs 1^st^ (lowest) | 1.02 (0.94-1.12) | 0.85 (0.77-0.94) | 0.99 (0.92-1.06) | 1.00 (0.94-1.07) |
|  | Number | 455,542 | 147,774 | 644,924 | 1,659,468 |

| **Table S6.** Race and ethnic associations between state-level negative sentiment and preterm birth by year | | | | |  |
| --- | --- | --- | --- | --- | --- |
| **Year** | **Data** | **Negative Black sentiment, birth outcomes among Black mothers** | **Negative Asian sentiment, birth outcomes among Asian mothers** | **Negative Latinx sentiment, birth outcomes among Latinx mothers** | **Negative White sentiment, birth outcomes among White mothers** |
| **2011** | 2nd tertile vs 1^st^ (lowest) | 1.04 (0.94-1.16) | 1.08 (0.99-1.18) | 0.98 (0.9-1.07) | 1.07 (1.01-1.13) |
|  | 3rd tertile vs 1^st^ (lowest) | 1.03 (0.91-1.16) | 1.02 (0.94-1.1) | 1.05 (0.94-1.17) | 1.04 (0.96-1.13) |
|  | Number | 423,325 | 207,701 | 756,251 | 1,680,812 |
| **2012** | 2nd tertile vs 1^st^ (lowest) | 1.13 (1.04-1.23) | 1.04 (0.96-1.13) | 1.07 (0.99-1.17) | 1.03 (0.96-1.1) |
|  | 3rd tertile vs 1^st^ (lowest) | 1.09 (1.01-1.17) | 1.06 (0.94-1.2) | 1.1 (1.02-1.18) | 1.09 (1.01-1.18) |
|  | Number | 442,579 | 229,358 | 759,011 | 1,729,525 |
| **2013** | 2nd tertile vs 1^st^ (lowest) | 1.10 (0.98-1.22) | 1.03 (0.95-1.12) | 1.08 (1.03-1.13) | 0.96 (0.9-1.02) |
|  | 3rd tertile vs 1^st^ (lowest) | 1.06 (0.95-1.18) | 1.03 (0.93-1.13) | 1.16 (1.08-1.24) | 0.97 (0.91-1.04) |
|  | Number | 461,682 | 231,050 | 754,239 | 1,765,632 |
| **2014** | 2nd tertile vs 1^st^ (lowest) | 1.09 (1.02-1.17) | 1.03 (0.96-1.1) | 0.98 (0.91-1.05) | 1.04 (0.99-1.1) |
|  | 3rd tertile vs 1^st^ (lowest) | 1.1 (1.01-1.2) | 1.12 (1.03-1.23) | 1.09 (0.99-1.19) | 1.08 (1.01-1.16) |
|  | Number | 471,311 | 211,547 | 805,769 | 1,860,703 |
| **2015** | 2nd tertile vs 1^st^ (lowest) | 1.14 (1.1-1.19) | 0.99 (0.93-1.06) | 0.98 (0.92-1.04) | 1.01 (0.93-1.09) |
|  | 3rd tertile vs 1^st^ (lowest) | 1.16 (1.1-1.22) | 1.11 (1.02-1.21) | 1.08 (0.99-1.18) | 1.01 (0.91-1.11) |
|  | Number | 493,345 | 218,687 | 837,242 | 1,896,892 |
| **2016** | 2nd tertile vs 1^st^ (lowest) | 1.13 (1.06-1.2) | 1.03 (0.97-1.09) | 0.9 (0.86-0.94) | 0.94 (0.88-1.0) |
|  | 3rd tertile vs 1^st^ (lowest) | 1.17 (1.09-1.25) | 1.02 (0.94-1.11) | 0.96 (0.9-1.03) | 0.95 (0.88-1.01) |
|  | Number | 499,662 | 235,108 | 853,805 | 1,905,676 |
| **2017** | 2nd tertile vs 1^st^ (lowest) | 1.07 (1.02-1.12) | 0.93 (0.86-1.0) | 0.91 (0.85-0.97) | 0.96 (0.91-1.01) |
|  | 3rd tertile vs 1^st^ (lowest) | 1.09 (1.04-1.14) | 1.01 (0.91-1.13) | 0.94 (0.88-1.0) | 0.99 (0.92-1.07) |
|  | Number | 485,364 | 165,056 | 631,915 | 1,735,764 |
| **2018** | 2nd tertile vs 1^st^ (lowest) | 1.08 (1.03-1.13) | 1.02 (0.94-1.1) | 0.98 (0.9-1.06) | 1.05 (0.97-1.13) |
|  | 3rd tertile vs 1^st^ (lowest) | 1.10 (1.04-1.15) | 1.06 (0.98-1.15) | 1.01 (0.94-1.09) | 0.99 (0.92-1.07) |
|  | Number | 478,703 | 160,640 | 627,420 | 1,708,235 |
| **2019** | 2nd tertile vs 1^st^ (lowest) | 1.03 (0.96-1.11) | 1.01 (0.93-1.1) | 0.97 (0.93-1.02) | 1.05 (0.97-1.15) |
|  | 3rd tertile vs 1^st^ (lowest) | 1.03 (0.96-1.12) | 1.02 (0.94-1.1) | 0.96 (0.9-1.02) | 1.02 (0.95-1.1) |
|  | Number | 478,902 | 159,893 | 635,824 | 1,680,757 |
| **2020** | 2nd tertile vs 1^st^ (lowest) | 1.09 (1.04-1.13) | 1.05 (0.96-1.16) | 1.03 (0.99-1.07) | 1.03 (0.95-1.12) |
|  | 3rd tertile vs 1^st^ (lowest) | 1.08 (1.03-1.13) | 1.01 (0.93-1.09) | 0.95 (0.9-1.0) | 0.99 (0.91-1.07) |
|  | Number | 466,890 | 150,944 | 629,155 | 1,623,755 |
| **2021** | 2nd tertile vs 1^st^ (lowest) | 1.10 (1.04-1.15) | 0.93 (0.85-1.01) | 0.99 (0.95-1.03) | 0.93 (0.87-0.99) |
|  | 3rd tertile vs 1^st^ (lowest) | 1.07 (1.01-1.13) | 0.90 (0.84-0.97) | 0.94 (0.88-1.01) | 0.91 (0.88-0.95) |
|  | Number | 455,837 | 147,824 | 645,205 | 1,660,159 |

| **Table S7.** Associations using incidence rate ratios between state-level racial sentiment towards minoritized and birth outcomes for full sample, minoritized mothers, and White mothers, 2011-2021, excluding users who tweeted more than 1,000 times per year | | | | |
| --- | --- | --- | --- | --- |
| **Year** | **Data** | **Total** | **Minoritized Groups** | **Whites** |
| **LBW** | 2nd tertile vs 1st (lowest) | 1.08 (1.02-1.14) | 1.09 (1.02-1.16) | 1.07 (1.01-1.14) |
|  | 3rd tertile | 1.09 (1.04-1.14) | 1.10 (1.04-1.16) | 1.09 (1.04-1.15) |
|  | N | 35,267,177 | 17,190,739 | 19,236,695 |
|  |  |  |  |  |
| **Preterm** | 2nd tertile vs 1st (lowest) | 1.06 (1.01-1.11) | 1.07 (1.02-1.12) | 1.06 (1.0-1.11) |
|  | 3rd tertile | 1.06 (1.01-1.12) | 1.07 (1.02-1.13) | 1.07 (1.0-1.14) |
|  | N | 35,288,014 | 17,200,907 | 19,247,910 |

| **Table S8**. Race and ethnic associations using incidence rate ratios between state-level negative sentiment and birth outcomes, 2011-2021, excluding users who tweeted more than 1,000 times per year | | | | | |
| --- | --- | --- | --- | --- | --- |
| Year | Data | Negative Black sentiment, birth outcomes among Black mothers | Negative Asian sentiment, birth outcomes among Asian mothers | Negative Latinx sentiment, birth outcomes among Black mothers | Negative White sentiment, birth outcomes among White mothers |
| **LBW** | 2nd tertile vs 1st (lowest) | 1.09 (1.03-1.15) | 0.99 (0.95-1.03) | 0.98 (0.95-1.01) | 1.00 (0.97-1.04) |
|  | 3rd tertile | 1.07 (1.02-1.12) | 1.01 (0.97-1.05) | 1.04 (1.0-1.09) | 1.00 (0.97-1.03) |
|  | N | 5,152,595 | 2,116,785 | 7,932,703 | 19,236,695 |
|  |  |  |  |  |  |
| **Preterm** | 2nd tertile vs 1st (lowest) | 1.10 (1.04-1.15) | 1.01 (0.97-1.05) | 0.98 (0.97-1.0) | 1.01 (0.98-1.04) |
|  | 3rd tertile | 1.09 (1.04-1.14) | 1.04 (0.99-1.08) | 1.02 (0.99-1.06) | 0.99 (0.95-1.03) |
|  | N | 5,157,600 | 2,117,808 | 7,935,836 | 19,247,910 |

| **Table S9.** Associations between county-level racial sentiment towards minoritized and birth outcomes for full sample, minoritized mothers, and White mothers, 2011-2021 | | | | |
| --- | --- | --- | --- | --- |
| **Year** | **Data** | **Total** | **Minoritized Groups** | **Whites** |
| **LBW** | 2nd tertile vs 1^st^ (lowest) | 1.00 (0.98-1.01) | 1.01 (0.99-1.04) | 0.99 (0.97-1.01) |
|  | 3rd tertile vs 1^st^ (lowest) | 1.02 (1.00-1.03) | 1.03 (1.01-1.06) | 1.00 (0.99-1.02) |
|  | N | 33,124,712 | 16,282,264 | 17,926,456 |
|  |  |  |  |  |
| **Preterm** | 2nd tertile vs 1^st^ (lowest) | 0.99 (0.97-1.01) | 0.99 (0.97-1.02) | 1.00 (0.98-1.01) |
|  | 3rd tertile vs 1^st^ (lowest) | 1.01 (0.99-1.03) | 1.02 (0.99-1.04) | 1.01 (0.99-1.02) |
|  | N | 33,144,765 | 16,292,149 | 17,937,154 |

| **Table S10.** Race and ethnic associations between county-level negative sentiment and birth outcomes, 2011-2021 | | | | | |
| --- | --- | --- | --- | --- | --- |
| **Year** | **Data** | **Negative Black sentiment, birth outcomes among Black mothers** | **Negative Asian sentiment, birth outcomes among Asian mothers** | **Negative Latinx sentiment, birth outcomes among Latinx mothers** | **Negative White sentiment, birth outcomes among White mothers** |
| **LBW** | 2nd tertile vs 1^st^ (lowest) | 1.01 (0.99-1.03) | 1.00 (0.97-1.04) | 0.98 (0.95-1.01) | 0.98 (0.96-0.99) |
|  | 3rd tertile vs 1^st^ (lowest) | 1.01 (0.99-1.03) | 1.01 (0.97-1.05) | 0.99 (0.97-1.02) | 0.98 (0.97-0.99) |
|  | N | 4,885,825 | 1,947,356 | 7,470,251 | 17,364,408 |
|  |  |  |  |  |  |
| **Preterm** | 2nd tertile vs 1^st^ (lowest) | 1.00 (0.98-1.02) | 0.95 (0.92-0.98) | 0.98 (0.95-1.0) | 0.97 (0.96-0.99) |
|  | 3rd tertile vs 1^st^ (lowest) | 1.01 (0.98-1.03) | 0.97 (0.94-1.0) | 0.99 (0.97-1.02) | 0.99 (0.98-1.0) |
|  | N | 4,890,690 | 1,948,350 | 7,473,250 | 17,374,841 |

| **Table S11.** Associations between county-level racial sentiment towards minoritized and low birth weight for full sample, minoritized mothers, and White mothers by year | | | | |
| --- | --- | --- | --- | --- |
| **Year** | **Data** | **Total** | **Minoritized Groups** | **Whites** |
| **2011** | 2nd tertile vs 1^st^ (lowest) | 1.00 (0.96-1.03) | 1.01 (0.97-1.06) | 0.98 (0.94-1.02) |
|  | 3rd tertile vs 1^st^ (lowest) | 1.02 (0.98-1.06) | 1.02 (0.97-1.08) | 1.01 (0.97-1.05) |
|  | Number | 2,944,645 | 1,425,993 | 1,611,422 |
| **2012** | 2nd tertile vs 1^st^ (lowest) | 1.02 (0.98-1.05) | 1.04 (1.0-1.09) | 0.99 (0.96-1.03) |
|  | 3rd tertile vs 1^st^ (lowest) | 1.03 (1.0-1.07) | 1.06 (1.01-1.12) | 1.01 (0.97-1.05) |
|  | Number | 3,075,393 | 1,481,028 | 1,692,702 |
| **2013** | 2nd tertile vs 1^st^ (lowest) | 1.01 (0.97-1.04) | 1.05 (1.0-1.1) | 0.99 (0.95-1.02) |
|  | 3rd tertile vs 1^st^ (lowest) | 1.02 (0.99-1.06) | 1.08 (1.03-1.13) | 0.99 (0.95-1.02) |
|  | Number | 3,130,243 | 1,499,788 | 1,732,000 |
| **2014** | 2nd tertile vs 1^st^ (lowest) | 1.00 (0.97-1.03) | 1.00 (0.96-1.05) | 1.00 (0.97-1.04) |
|  | 3rd tertile vs 1^st^ (lowest) | 1.03 (1.0-1.06) | 1.05 (1.0-1.1) | 1.03 (0.99-1.06) |
|  | Number | 3,368,733 | 1,650,222 | 1,822,440 |
| **2015** | 2nd tertile vs 1^st^ (lowest) | 0.98 (0.95-1.02) | 0.98 (0.94-1.03) | 0.99 (0.96-1.02) |
|  | 3rd tertile vs 1^st^ (lowest) | 1.00 (0.97-1.04) | 1.00 (0.96-1.05) | 1.01 (0.98-1.05) |
|  | Number | 3,408,349 | 1,708,044 | 1,812,017 |
| **2016** | 2nd tertile vs 1^st^ (lowest) | 1.00 (0.97-1.03) | 1.01 (0.97-1.05) | 1.00 (0.97-1.03) |
|  | 3rd tertile vs 1^st^ (lowest) | 1.02 (0.99-1.05) | 1.04 (0.99-1.09) | 1.00 (0.97-1.03) |
|  | Number | 3,456,121 | 1,753,623 | 1,819,765 |
| **2017** | 2nd tertile vs 1^st^ (lowest) | 1.01 (0.98-1.03) | 1.03 (1.0-1.07) | 1.00 (0.97-1.03) |
|  | 3rd tertile vs 1^st^ (lowest) | 1.02 (0.99-1.04) | 1.04 (1.0-1.08) | 1.02 (0.98-1.05) |
|  | Number | 2,966,750 | 1,417,331 | 1,648,664 |
| **2018** | 2nd tertile vs 1^st^ (lowest) | 0.98 (0.96-1.01) | 1.00 (0.96-1.03) | 0.97 (0.94-0.99) |
|  | 3rd tertile vs 1^st^ (lowest) | 0.99 (0.96-1.01) | 1.01 (0.97-1.05) | 0.97 (0.94-1.0) |
|  | Number | 2,928,604 | 1,402,152 | 1,623,458 |
| **2019** | 2nd tertile vs 1^st^ (lowest) | 0.98 (0.95-1.01) | 0.99 (0.96-1.03) | 0.97 (0.94-1.0) |
|  | 3rd tertile vs 1^st^ (lowest) | 0.99 (0.96-1.02) | 1.01 (0.97-1.04) | 0.98 (0.95-1.01) |
|  | Number | 2,908,320 | 1,409,386 | 1,593,554 |
| **2020** | 2nd tertile vs 1^st^ (lowest) | 1.02 (1.0-1.05) | 1.04 (1.01-1.07) | 1.01 (0.98-1.03) |
|  | 3rd tertile vs 1^st^ (lowest) | 1.05 (1.02-1.08) | 1.05 (1.02-1.09) | 1.05 (1.02-1.08) |
|  | Number | 2,830,892 | 1,380,558 | 1,539,746 |
| **2021** | 2nd tertile vs 1^st^ (lowest) | 0.99 (0.95-1.03) | 0.99 (0.95-1.03) | 1.00 (0.95-1.05) |
|  | 3rd tertile vs 1^st^ (lowest) | 1.00 (0.96-1.04) | 0.99 (0.95-1.05) | 1.02 (0.96-1.08) |
|  | Number | 2,106,662 | 1,154,139 | 1,030,688 |

| **Table S12.** Associations between county-level racial sentiment towards minoritized and preterm birth for full sample, minoritized mothers, and White mothers by year | | | | |
| --- | --- | --- | --- | --- |
| **Year** | **Data** | **Total** | **Minoritized Groups** | **Whites** |
| **2011** | 2nd tertile vs 1^st^ (lowest) | 0.99 (0.97-1.03) | 1.00 (0.96-1.04) | 0.99 (0.96-1.02) |
|  | 3rd tertile vs 1^st^ (lowest) | 1.02 (0.99-1.05) | 1.01 (0.97-1.06) | 1.02 (0.99-1.06) |
|  | Number | 2,946,426 | 1,426,871 | 1,612,366 |
| **2012** | 2nd tertile vs 1^st^ (lowest) | 1.02 (0.99-1.05) | 1.03 (0.99-1.07) | 1.01 (0.99-1.04) |
|  | 3rd tertile vs 1^st^ (lowest) | 1.03 (1.0-1.07) | 1.04 (1.0-1.09) | 1.02 (0.99-1.06) |
|  | Number | 3,077,575 | 1,482,132 | 1,693,847 |
| **2013** | 2nd tertile vs 1^st^ (lowest) | 1.00 (0.97-1.03) | 1.01 (0.96-1.05) | 1.00 (0.98-1.03) |
|  | 3rd tertile vs 1^st^ (lowest) | 1.00 (0.97-1.03) | 1.01 (0.96-1.06) | 1.00 (0.97-1.03) |
|  | Number | 3,132,497 | 1,500,943 | 1,733,149 |
| **2014** | 2nd tertile vs 1^st^ (lowest) | 1.01 (0.98-1.04) | 1.01 (0.96-1.06) | 1.02 (0.99-1.04) |
|  | 3rd tertile vs 1^st^ (lowest) | 1.01 (0.98-1.04) | 1.01 (0.96-1.06) | 1.02 (0.99-1.05) |
|  | Number | 3,370,377 | 1,650,990 | 1,823,346 |
| **2015** | 2nd tertile vs 1^st^ (lowest) | 1.00 (0.97-1.03) | 0.99 (0.94-1.04) | 1.01 (0.98-1.04) |
|  | 3rd tertile vs 1^st^ (lowest) | 1.01 (0.97-1.04) | 0.99 (0.94-1.05) | 1.02 (0.99-1.05) |
|  | Number | 3,410,432 | 1,708,958 | 1,813,256 |
| **2016** | 2nd tertile vs 1^st^ (lowest) | 0.99 (0.97-1.02) | 1.00 (0.97-1.03) | 0.99 (0.96-1.02) |
|  | 3rd tertile vs 1^st^ (lowest) | 1.01 (0.99-1.04) | 1.04 (1.0-1.08) | 0.99 (0.97-1.02) |
|  | Number | 3,458,990 | 1,754,964 | 1,821,377 |
| **2017** | 2nd tertile vs 1^st^ (lowest) | 0.99 (0.97-1.02) | 1.01 (0.97-1.04) | 0.99 (0.97-1.02) |
|  | 3rd tertile vs 1^st^ (lowest) | 1.02 (0.99-1.04) | 1.04 (1.0-1.07) | 1.01 (0.98-1.04) |
|  | Number | 2,968,262 | 1,418,108 | 1,649,439 |
| **2018** | 2nd tertile vs 1^st^ (lowest) | 0.96 (0.94-0.99) | 0.97 (0.93-1.02) | 0.96 (0.94-0.99) |
|  | 3rd tertile vs 1^st^ (lowest) | 0.99 (0.96-1.02) | 1.00 (0.95-1.05) | 0.99 (0.96-1.02) |
|  | Number | 2,930,031 | 1,402,817 | 1,624,252 |
| **2019** | 2nd tertile vs 1^st^ (lowest) | 0.98 (0.95-1.01) | 0.99 (0.95-1.03) | 0.98 (0.96-1.01) |
|  | 3rd tertile vs 1^st^ (lowest) | 1.00 (0.98-1.03) | 1.01 (0.96-1.05) | 1.01 (0.98-1.04) |
|  | Number | 2,910,199 | 1,410,332 | 1,594,550 |
| **2020** | 2nd tertile vs 1^st^ (lowest) | 0.99 (0.97-1.02) | 1.00 (0.96-1.04) | 0.99 (0.97-1.02) |
|  | 3rd tertile vs 1^st^ (lowest) | 1.02 (0.99-1.05) | 1.02 (0.98-1.06) | 1.02 (0.99-1.05) |
|  | Number | 2,832,321 | 1,381,304 | 1,540,456 |
| **2021** | 2nd tertile vs 1^st^ (lowest) | 0.98 (0.94-1.03) | 0.98 (0.93-1.03) | 1.00 (0.95-1.06) |
|  | 3rd tertile vs 1^st^ (lowest) | 0.99 (0.95-1.04) | 0.98 (0.93-1.04) | 1.02 (0.96-1.08) |
|  | Number | 2,107,655 | 1,154,730 | 1,031,116 |

| **Table S13.** Race and ethnic associations between county-level negative sentiment and low birth weight by year | | | | | |
| --- | --- | --- | --- | --- | --- |
| **Year** | **Data** | **Negative Black sentiment, birth outcomes among Black mothers** | **Negative Asian sentiment, birth outcomes among Asian mothers** | **Negative Latinx sentiment, birth outcomes among Latinx mothers** | **Negative White sentiment, birth outcomes among White mothers** |
| 2011 | 2nd tertile vs 1^st^ (lowest) | 0.98 (0.94-1.02) | 1.08 (0.99-1.18) | 0.97 (0.89-1.06) | 0.95 (0.91-0.99) |
|  | 3rd tertile vs 1^st^ (lowest) | 0.98 (0.93-1.03) | 1.07 (1.0-1.14) | 1.00 (0.95-1.05) | 0.96 (0.93-1.0) |
|  | Number | 408,354 | 187,808 | 702,025 | 1,476,145 |
| 2012 | 2nd tertile vs 1^st^ (lowest) | 1.03 (0.94-1.13) | 0.97 (0.87-1.09) | 0.95 (0.88-1.03) | 0.97 (0.94-1.01) |
|  | 3rd tertile vs 1^st^ (lowest) | 1.03 (0.93-1.13) | 0.96 (0.86-1.08) | 0.97 (0.9-1.03) | 0.96 (0.93-0.99) |
|  | Number | 428,155 | 213,353 | 719,999 | 1,642,915 |
| 2013 | 2nd tertile vs 1^st^ (lowest) | 1.00 (0.95-1.05) | 1.07 (0.97-1.18) | 0.98 (0.92-1.05) | 0.97 (0.95-1.0) |
|  | 3rd tertile vs 1^st^ (lowest) | 0.98 (0.93-1.04) | 1.12 (1.02-1.23) | 1.01 (0.96-1.07) | 0.98 (0.95-1.01) |
|  | Number | 447,616 | 214,588 | 718,231 | 1,711,110 |
| 2014 | 2nd tertile vs 1^st^ (lowest) | 1.04 (0.98-1.09) | 1.07 (0.93-1.23) | 0.98 (0.92-1.04) | 0.98 (0.95-1.02) |
|  | 3rd tertile vs 1^st^ (lowest) | 1.06 (1.01-1.12) | 1.09 (0.94-1.27) | 1.00 (0.94-1.07) | 1.00 (0.97-1.03) |
|  | Number | 459,092 | 198,439 | 779,098 | 1,791,352 |
| 2015 | 2nd tertile vs 1^st^ (lowest) | 0.99 (0.95-1.05) | 0.9 (0.81-1.01) | 0.96 (0.9-1.03) | 0.97 (0.95-1.0) |
|  | 3rd tertile vs 1^st^ (lowest) | 1.02 (0.97-1.07) | 1.01 (0.92-1.11) | 0.97 (0.92-1.03) | 0.99 (0.96-1.02) |
|  | Number | 474,936 | 205,033 | 806,566 | 1,773,071 |
| 2016 | 2nd tertile vs 1^st^ (lowest) | 1.01 (0.95-1.06) | 0.92 (0.83-1.01) | 0.97 (0.91-1.03) | 0.97 (0.94-0.99) |
|  | 3rd tertile vs 1^st^ (lowest) | 1.01 (0.95-1.07) | 0.86 (0.78-0.94) | 0.96 (0.9-1.03) | 0.97 (0.94-1.0) |
|  | Number | 479,980 | 220,025 | 824,275 | 1,781,813 |
| 2017 | 2nd tertile vs 1^st^ (lowest) | 1.02 (0.98-1.07) | 0.96 (0.89-1.04) | 1.04 (0.99-1.09) | 0.98 (0.95-1.01) |
|  | 3rd tertile vs 1^st^ (lowest) | 1.01 (0.96-1.07) | 0.99 (0.91-1.07) | 1.02 (0.97-1.07) | 0.96 (0.93-0.99) |
|  | Number | 464,809 | 150,608 | 600,775 | 1,591,540 |
| 2018 | 2nd tertile vs 1^st^ (lowest) | 1.01 (0.98-1.04) | 0.89 (0.81-0.98) | 1.02 (0.96-1.09) | 0.97 (0.95-1.0) |
|  | 3rd tertile vs 1^st^ (lowest) | 1.02 (0.98-1.06) | 0.91 (0.83-1.01) | 1.03 (0.97-1.09) | 0.99 (0.96-1.02) |
|  | Number | 459,396 | 146,969 | 597,361 | 1,565,299 |
| 2019 | 2nd tertile vs 1^st^ (lowest) | 1.00 (0.97-1.04) | 0.95 (0.86-1.04) | 0.98 (0.93-1.03) | 0.98 (0.95-1.01) |
|  | 3rd tertile vs 1^st^ (lowest) | 1.01 (0.96-1.05) | 0.94 (0.86-1.03) | 0.98 (0.93-1.04) | 0.99 (0.96-1.03) |
|  | Number | 459,239 | 146,433 | 603,679 | 1,531,669 |
| 2020 | 2nd tertile vs 1^st^ (lowest) | 1.05 (1.01-1.1) | 1.08 (0.96-1.21) | 0.99 (0.94-1.05) | 0.99 (0.96-1.02) |
|  | 3rd tertile vs 1^st^ (lowest) | 1.05 (1.0-1.1) | 1.09 (0.96-1.23) | 1.01 (0.96-1.06) | 1.01 (0.98-1.04) |
|  | Number | 448,511 | 138,869 | 595,907 | 1,477,868 |
| 2021 | 2nd tertile vs 1^st^ (lowest) | 1.02 (0.96-1.08) | 0.97 (0.84-1.12) | 1.05 (0.96-1.15) | 1.04 (0.99-1.09) |
|  | 3rd tertile vs 1^st^ (lowest) | 0.98 (0.91-1.05) | 0.94 (0.81-1.08) | 1.05 (0.97-1.14) | 1.04 (0.98-1.1) |
|  | Number | 355,737 | 125,231 | 522,335 | 1,021,626 |

| **Table S14.** Race and ethnic associations between county-level negative sentiment and preterm birth by year | | | | |  |
| --- | --- | --- | --- | --- | --- |
| **Year** | **Data** | **Negative Black sentiment, birth outcomes among Black mothers** | **Negative Asian sentiment, birth outcomes among Asian mothers** | **Negative Latinx sentiment, birth outcomes among Latinx mothers** | **Negative White sentiment, birth outcomes among White mothers** |
| **2011** | 2nd tertile vs 1^st^ (lowest) | 0.95 (0.9-1.0) | 1.03 (0.94-1.12) | 0.98 (0.9-1.06) | 0.98 (0.95-1.01) |
|  | 3rd tertile vs 1^st^ (lowest) | 0.95 (0.9-1.01) | 1.00 (0.94-1.07) | 0.98 (0.94-1.03) | 0.99 (0.96-1.02) |
|  | Number | 408,839 | 187,886 | 702,282 | 1,477,016 |
| **2012** | 2nd tertile vs 1^st^ (lowest) | 1.03 (0.89-1.2) | 0.95 (0.87-1.04) | 0.99 (0.93-1.04) | 0.97 (0.95-1.0) |
|  | 3rd tertile vs 1^st^ (lowest) | 1.04 (0.89-1.22) | 0.96 (0.88-1.04) | 1.01 (0.96-1.06) | 0.95 (0.92-0.98) |
|  | Number | 428,794 | 213,457 | 720,285 | 1,644,031 |
| **2013** | 2nd tertile vs 1^st^ (lowest) | 0.93 (0.87-1.0) | 0.92 (0.85-1.0) | 0.98 (0.93-1.03) | 0.98 (0.95-1.0) |
|  | 3rd tertile vs 1^st^ (lowest) | 0.93 (0.87-1.0) | 1.00 (0.92-1.09) | 1.02 (0.97-1.07) | 1.00 (0.97-1.02) |
|  | Number | 448,292 | 214,718 | 718,526 | 1,712,254 |
| **2014** | 2nd tertile vs 1^st^ (lowest) | 1.01 (0.95-1.07) | 0.95 (0.84-1.08) | 0.97 (0.91-1.03) | 0.99 (0.96-1.01) |
|  | 3rd tertile vs 1^st^ (lowest) | 1.01 (0.95-1.07) | 0.99 (0.87-1.13) | 1.00 (0.95-1.06) | 1.01 (0.99-1.04) |
|  | Number | 459,485 | 198,491 | 779,329 | 1,792,244 |
| **2015** | 2nd tertile vs 1^st^ (lowest) | 1.00 (0.95-1.06) | 0.92 (0.83-1.02) | 0.96 (0.92-1.01) | 0.98 (0.95-1.0) |
|  | 3rd tertile vs 1^st^ (lowest) | 1.02 (0.97-1.08) | 1.01 (0.92-1.11) | 1.00 (0.96-1.05) | 1.01 (0.98-1.03) |
|  | Number | 475,348 | 205,132 | 806,831 | 1,774,299 |
| **2016** | 2nd tertile vs 1^st^ (lowest) | 1.01 (0.96-1.07) | 0.87 (0.8-0.95) | 0.95 (0.89-1.01) | 0.95 (0.93-0.98) |
|  | 3rd tertile vs 1^st^ (lowest) | 1.02 (0.97-1.08) | 0.87 (0.8-0.95) | 0.95 (0.9-1.0) | 0.98 (0.95-1.01) |
|  | Number | 480,591 | 220,207 | 824,679 | 1,783,398 |
| **2017** | 2nd tertile vs 1^st^ (lowest) | 0.99 (0.94-1.04) | 0.97 (0.87-1.07) | 0.99 (0.94-1.05) | 0.96 (0.94-0.99) |
|  | 3rd tertile vs 1^st^ (lowest) | 1.01 (0.95-1.07) | 0.96 (0.86-1.06) | 1.00 (0.95-1.06) | 0.98 (0.95-1.01) |
|  | Number | 465,162 | 150,691 | 601,026 | 1,592,286 |
| **2018** | 2nd tertile vs 1^st^ (lowest) | 0.99 (0.95-1.04) | 0.91 (0.83-1.01) | 1.01 (0.96-1.07) | 0.97 (0.94-1.0) |
|  | 3rd tertile vs 1^st^ (lowest) | 1.00 (0.95-1.05) | 0.94 (0.85-1.04) | 1.01 (0.95-1.07) | 0.99 (0.96-1.02) |
|  | Number | 459,730 | 147,023 | 597,562 | 1,566,075 |
| **2019** | 2nd tertile vs 1^st^ (lowest) | 1.01 (0.97-1.05) | 0.9 (0.81-0.99) | 1.02 (0.96-1.07) | 0.97 (0.94-1.0) |
|  | 3rd tertile vs 1^st^ (lowest) | 1.01 (0.96-1.06) | 0.89 (0.81-0.98) | 0.99 (0.94-1.05) | 0.97 (0.95-1.01) |
|  | Number | 459,629 | 146,546 | 603,998 | 1,532,643 |
| **2020** | 2nd tertile vs 1^st^ (lowest) | 1.04 (0.99-1.09) | 0.91 (0.78-1.06) | 0.99 (0.94-1.04) | 0.98 (0.95-1.0) |
|  | 3rd tertile vs 1^st^ (lowest) | 1.06 (1.01-1.11) | 0.95 (0.81-1.1) | 0.99 (0.94-1.03) | 1.00 (0.97-1.03) |
|  | Number | 448,843 | 138,923 | 596,168 | 1,478,542 |
| **2021** | 2nd tertile vs 1^st^ (lowest) | 1.01 (0.96-1.07) | 1.05 (0.9-1.24) | 1.02 (0.94-1.11) | 1.00 (0.93-1.06) |
|  | 3rd tertile vs 1^st^ (lowest) | 0.99 (0.92-1.06) | 1.01 (0.85-1.2) | 1.04 (0.96-1.12) | 1.01 (0.94-1.08) |
|  | Number | 355,977 | 125,276 | 522,564 | 1,022,053 |

**Example code**

**Step 1: Historical Tweets Request Example (Jupyter Notebook)**

# import packages

import logging

import time

import requests

import os

import json

import pandas as pd

import csv

import datetime

import dateutil.parser

import unicodedata

import time

import multiprocessing as mp

import math

import requests

from itertools import cycle

from lxml.html import fromstring

from itertools import cycle

import numpy as np

import tqdm

from tqdm import tqdm

import gc

import gzip

import json

import sys

tqdm.pandas()

from datetime import datetime as dtime

# put your result path here

collect_dir = "./"

# put your token here, it can take multiple tokens

bearer_tokens = [

'token1',

'token2',

'token3'

]

# create headers for API

def create_headers(bearer_token):

headers = {"Authorization": "Bearer {}".format(bearer_token),

"Cache-Control":"no-cache"}

return headers

# error class

class TooManyRequests(Exception):

def __init__(self, message):

super().__init__(message)

class ChangeBearerToken(Exception):

def __init__(self, message):

super().__init__(message)

class GeneralException(Exception):

def __init__(self, message):

super().__init__(message)

class ServiceNotAvailable(Exception):

def __init__(self, message):

super().__init__(message)

def connect_to_endpoint(url, headers, params, next_token = None):

global response

params['next_token'] = next_token

time.sleep(3)

response = requests.request("GET", url, headers = headers, params = params)

if response.status_code != 200:

if "TooManyRequests" in response.text:

raise TooManyRequests(response.text)

elif "UsageCapExceeded" in response.text:

raise ChangeBearerToken(response.text)

elif "Service Unavailable" in response.text or response.status_code == 503:

raise ServiceNotAvailable(response.text)

else:

raise GeneralException(response.text)

return response.json()

# results will be saved as gz files in a monthly folder

def append_to_gz(merged_dicts, fileName):

fout = gzip.open(fileName, "a")

try:

json_str = json.dumps(merged_dicts) + "

"

json_bytes = json_str.encode('utf-8')

fout.write(json_bytes)

except IndexError:

print("Tweets Written Failed")

fout.close()

# function to request tweets

def get_tweets(bearer_token, folder_name, fname, startdate, enddate):

global json_response;

global response

global merged_dicts;

headers = create_headers(bearer_token)

max_results = 500 # define the max results that one page can collect. Change to a smaller number will increase the speed for collecting a page data, but will result in needing more pages.

url = "https://api.twitter.com/2/tweets/search/all"

# change this query params based on your own needs; you can define keywords/location/time etc.

# for more information including limits, please read the official twitter api documents.

query_params = {"query": "(asian OR Japanese OR thai OR korean) place_country:US has:geo lang:en",

'max_results': max_results,

'start_time': startdate,

'end_time': enddate,

'expansions': 'geo.place_id,author_id,in_reply_to_user_id,referenced_tweets.id,referenced_tweets.id.author_id,entities.mentions.username',

'tweet.fields': 'id,text,author_id,created_at,geo,lang,public_metrics,source,conversation_id,in_reply_to_user_id,referenced_tweets',

'user.fields': 'id,name,username,created_at,description,location,pinned_tweet_id,public_metrics,verified',

'place.fields': 'full_name,id,geo,name,place_type',

'next_token':{}}

total_tweets = 0

flag = True

next_token = None

page = 0;

print("Start time: " + str(startdate) + " Current time: " + str(dtime.now()))

while flag:

page = page + 1;

json_response = connect_to_endpoint(url, headers, query_params, next_token)

print("

Page #: " + str(page), end=". ")

print("Tweets Received: " + str(len(json_response['data'])), end=". ")

append_to_gz(json_response, collect_dir + folder_name + '/' + fname +".gz")

while True:

if 'next_token' in json_response['meta']:

next_token = json_response['meta']['next_token']

else:

flag = False

next_token = None

break

total_tweets = total_tweets + len(json_response['data'])

print("Total number of results: ", str(total_tweets))

# note that for the listed error exceptions here, the current page will be restarted, which means duplicate tweets may be collected. Remove duplicates during the post process.

def do_everything(folder_name, fname, start, end, iii):

iii = iii

while True:

try:

if not os.path.isdir(collect_dir + folder_name):

os.makedirs(collect_dir + folder_name)

get_tweets(bearer_tokens[iii], folder_name, fname, start, end)

except ChangeBearerToken as e:

print('Change bearer token: ' + str(e) + ' restart this page')

iii = iii + 1

if iii == len(bearer_tokens):

iii = 0

print("All tokens are used for current time period")

continue

except GeneralException as e:

print('General exception: ' + str(e) + ' restart this page')

continue

except TooManyRequests as e:

print('Too many requests: ' + str(e) + ' restart this page')

time.sleep(10)

continue

except ServiceNotAvailable as e:

print('Service not available: ' + str(e) + ' restart this page')

continue

break

return iii

# put the timeframe you want to collect tweets from.

date = pd.date_range(start='2022-01-01T00:00:00.000Z', periods=745, freq='H')

date = [datetime.datetime.strftime(i, "%Y-%m-%dT%H:00:00Z") for i in date]

# main code

iii = 0

for i in range(len(date) - 1):

connected = False

while not connected:

try:

start_time = date[i];

end_time = date[i + 1];

iii = do_everything( start_time[:7], start_time[8:10], start_time, end_time, iii)

print("current bearer token is " + str(iii))

connected = True

except Exception as e:

print('figure out why' + str(e) + "*********************")

# date-hour that with issues will be saved to a file.

with open ("C:/Users/xyue/collected_data_full_academic/log_witherros.csv",'a', newline='') as filedata:

writer = csv.writer(filedata)

row = [str(start_time)]

writer.writerow(row)

filedata.close()

connected = True

**Step 2: Historical Tweets Filtering Example (Python Programming Language)**

import argparse

import copy

import glob

import gzip

import json

import sys

import re

import os

import time

import csv

import datetime

# there are some limits when using key word to collect tweets.

# for example, a key word term "white boy" will match with the tweet "white shirt boy".

# so this file is used to filter out irrelevant tweets and also convert gz files to csv files.

# define path

# set key word list path

infile="E:/key_word_list.csv"

# define target files

Mon="*.gz"

indir = "E:/" + Mon

# set result file path

outfile= "E:/" + "result.csv"

# process keyword list

def processKeywords(infile):

raceDict = {}

with open(infile, 'r', encoding="utf-8") as g:

print("Processing file: {}".format(infile))

g.readline() #really should process with csv library. maybe later

for line in g:

pieces = [x.lower().strip() for x in line.split(",")]

try:

assert pieces[0] not in raceDict

except AssertionError:

print("{} is a duplicate".format(pieces[0]))

continue

if pieces[1]=="exclude":

continue

raceDict[pieces[0]] = {"Race":pieces[1]}

assert " " not in raceDict

print("raceDict has {} entries".format(len(raceDict)))

return(raceDict)

# process tweets

# find and extract parameters based on needs

def processTweets(indir, outfile, raceDict):

OUTFILE = open(outfile, "w", encoding='utf-8', newline='')

labels = ["conversation_id", "tweet_id", "tweet_text", "tweet_timestamp",

"geo_coord", "place_id", "place_bbox", "place_full_name",

"place_type", "place_name", "entities_tweet_mention_username",

"entities_tweet_mention_id", "user_id", "user_place", "user_name",

"user_username", "user_create_at", "user_profile", "user_pinned_tweet_id",

"user_follower_count", "user_following_count", "user_tweets_count",

"user_listed_count", "user_verified", "tweets_in_reply_to_user_id", "tweets_language",

"tweets_retweet_count", "tweets_reply_count", "tweets_like_count",

"tweets_quote_count", "tweets_referenced_type", "tweets_referenced_id",

"tweets_source", "racecat1", "racecat2", "racecat3", "raceterm1",

"raceterm2", "raceterm3" ]

writer = csv.writer(OUTFILE)

writer.writerow(labels)

decodeErrors = 0

valueErrors = 0

typeKeyErrors = 0

noErrors = 0

totalFinds = 0

raceList = []

racePairList = []

for k in raceDict:

if k.startswith("#"):

s = r'%s\b' % k #for hashtags, just check boundary on right

raceList.append(s)

elif "+" not in k:

s = r'\b%s\b' % k

raceList.append(s)

else:

pieces = [x.strip() for x in k.split("+")]

piece1 = r'\b%s\b' % pieces[0]

piece2 = r'\b%s\b' % pieces[1]

racePairList.append([piece1, piece2])

raceRegexS = "|".join(raceList)

raceBigRegex = re.compile(raceRegexS)

for fname in glob.glob(indir):

print("Working on file: {}".format(fname))

print(datetime.datetime.now())

if not fname.endswith(".gz"):

print("I expect all files to end with .gz. Skipping file {}".format(fname))

continue

for line in gzip.open(fname):

if not line:

continue

try:

line = line.decode('utf-8').strip()

except UnicodeDecodeError:

decodeErrors +=1

continue

try:

tweet_obj = json.loads(line, encoding='utf-8')

except ValueError:

# Skip lines that aren't JSON objects

# e.g., '[WARN]' lines

valueErrors +=1

continue

for i in range(len(tweet_obj['data'])):

#noErrors count number of general tweets collected

noErrors +=1

#get tweet text

origTweet = tweet_obj['data'][i]['text']

#get rid of troublesome white space

origTweet = origTweet.replace("\t", " ")

origTweet = origTweet.replace("\n", " ")

origTweet = origTweet.replace("\r", " ")

origTweet = re.sub(r"\s+", ' ', origTweet)

tweet = origTweet.lower() #use this for searching but keep orig

#hopefully this speeds things up because it's one search instead of 500+ for most things

racePair = False

for pair in racePairList:

n = re.search(pair[0], tweet)

p = re.search(pair[1], tweet)

if n is not None and p is not None:

racePair = True

break

m = re.search(raceBigRegex, tweet)

if m is None and racePair==False:

continue

finds = [] #all matches

for k in raceDict: #search for every term in the dictionary

if k.startswith("#"):

searchTerm = r'%s\b' % k #just check for boundary on right

m = re.finditer(searchTerm, tweet)

for match in m:

finds.append(match.span())#keep index in sentence of finds

elif "+" not in k: # '+' search terms are different

searchTerm = r'\b%s\b' % k

m = re.finditer(searchTerm, tweet)

for match in m:

finds.append(match.span()) #keep index in sentence of finds

else:

search1, search2 = [x.strip() for x in k.split("+")] #eg tamir + rice

searchTerm1 = r'\b%s\b' % search1

searchTerm2 = r'\b%s\b' % search2

m = re.search(searchTerm1, tweet)

n = re.search(searchTerm2, tweet)

if m is not None and n is not None:

finds.append(k)

#warning, don't forget, maybe change in future, variable finds can

#contain 2 types of data, a span e.g. (4, 10) or a string e.g.

#"tamir + rice"!!!

if len(finds)==0:

continue #no matches, continue to next Tweet

if len(finds)>3:

continue #ignoring those that have more than 3 race terms

info = []

#1. conversationID

try:

info.append(str(tweet_obj['data'][i]['conversation_id'])+'g')

except:

convs_id = ''

info.append(convs_id)

#2. tweetID

info.append(str(tweet_obj['data'][i]['id'])+'g')

#3. tweet text

info.append(origTweet)

#4. timestamp

info.append(tweet_obj['data'][i]["created_at"])

#5. lat/long

latlon = ''

if "geo" in tweet_obj['data'][i].keys():

if "coordinates" in tweet_obj['data'][i]['geo'].keys():

if str(tweet_obj['data'][i]['geo']['coordinates']['type']) == "Point":

latlon = tweet_obj['data'][i]['geo']['coordinates']['coordinates']

else:

latlon = ''

else:

latlon = ''

info.append(str(latlon))

#place field (about tweet)

#6. place id

#7. bbox

#8. full name

#9. place type

#10. place name

#11. entities tweet mention username

#12. entites tweet mention id

bb = ''

ptl = ''

pty = ''

pname = ''

p_id = ''

tm = ''

tmid = ''

if 'geo' in tweet_obj['data'][i].keys():

if 'place_id' in tweet_obj['data'][i]['geo'].keys():

p_id = tweet_obj['data'][i]['geo']['place_id']

for p in range(len(tweet_obj['includes']['places'])):

if tweet_obj['includes']['places'][p]['id'] == p_id:

if 'geo' in tweet_obj['includes']['places'][p].keys():

if str(tweet_obj['includes']['places'][p]['geo']['type']) == "Feature":

bb = tweet_obj['includes']['places'][p]['geo']['bbox']

if 'full_name' in tweet_obj['includes']['places'][p].keys():

ptl = tweet_obj['includes']['places'][p]['full_name'].replace(",", ";")

if 'place_type' in tweet_obj['includes']['places'][p].keys():

pty = tweet_obj['includes']['places'][p]['place_type']

if 'name' in tweet_obj['includes']['places'][p].keys():

pname = tweet_obj['includes']['places'][p]['name']

break

if 'entities' in tweet_obj['data'][i].keys():

if 'mentions' in str(tweet_obj['data'][i]['entities'].keys()):

if 'username' in str(tweet_obj['data'][i]['entities']['mentions'][0].keys()):

tm = str(tweet_obj['data'][i]['entities']['mentions'][0]['username'])

if 'id' in str(tweet_obj['data'][i]['entities']['mentions'][0].keys()):

tmid = str(tweet_obj['data'][i]['entities']['mentions'][0]['id']) + "g"

info.append(str(p_id))

info.append(str(bb))

info.append(str(ptl))

info.append(str(pty))

info.append(str(pname))

info.append(str(tm))

info.append(str(tmid))

# user field

pal = ''

us = ''

nf = ''

nam = ''

crt = ''

descp = ''

ptid = ''

nfing = ''

nt = ''

nl = ''

verif = ''

descp_clean = ''

nam_clean = ''

pal_clean = ''

a_id = ''

if 'author_id' in tweet_obj['data'][i].keys():

a_id = tweet_obj['data'][i]['author_id']

for a in range(len(tweet_obj['includes']['users'])):

if tweet_obj['includes']['users'][a]['id'] == a_id:

if 'location' in tweet_obj['includes']['users'][a].keys():

pal = str(tweet_obj['includes']['users'][a]['location'].replace(",", ";"))

pal_clean = re.sub(r"\s+", ' ', pal)

if 'pinned_tweet_id' in tweet_obj['includes']['users'][a].keys():

ptid = str(tweet_obj['includes']['users'][a]['pinned_tweet_id']) + "g"

try:

nam = tweet_obj['includes']['users'][a]['name']

nam_clean = re.sub(r"\s+", ' ', nam)

except:

nam_clean = ''

try:

us = tweet_obj['includes']['users'][a]['username']

except:

us = ''

try:

crt = tweet_obj['includes']['users'][a]['created_at']

except:

crt = ''

try:

descp = tweet_obj['includes']['users'][a]['description']

descp_clean = re.sub(r"\s+", ' ', descp)

except:

descp_clean = ''

try:

nf = tweet_obj['includes']['users'][a]['public_metrics']['followers_count']

except:

nf = ''

try:

nfing = tweet_obj['includes']['users'][a]['public_metrics']['following_count']

except:

nfing = ''

try:

nt = tweet_obj['includes']['users'][a]['public_metrics']['tweet_count']

except:

nt = ''

try:

nl = tweet_obj['includes']['users'][a]['public_metrics']['listed_count']

except:

nl = ''

try:

verif = tweet_obj['includes']['users'][a]['verified']

except:

verif = ''

break

#13. author id

#14. place_account_level

#15. name

#16. username

#17. create at

#18. description

#19. pinned tweet id

#20. number of followers

#21. number of following

#22. number of tweets

#23. number of listed

#24. user is verified or not

info.append(str(a_id)+'g')

info.append(str(pal_clean))

info.append(str(nam_clean))

info.append(str(us))

info.append(str(crt))

info.append(str(descp_clean))

info.append(str(ptid))

info.append(str(nf))

info.append(str(nfing))

info.append(str(nt))

info.append(str(nl))

info.append(str(verif))

#25. tweet: in reply to user id

#26. tweet language

#27. tweet retweet count

#28. tweet reply count

#29. tweet like count

#30. tweet quote count

#31. referenced_tweets type

#32. referenced_tweets id

#33. source

irtuid = ''

tl = ''

retc = ''

repc = ''

likc = ''

quotc = ''

rftt = ''

rftid = ''

src = ''

if 'lang' in tweet_obj['data'][i].keys():

tl = str(tweet_obj['data'][i]['lang'])

if 'source' in tweet_obj['data'][i].keys():

src = str(tweet_obj['data'][i]['source'])

if 'referenced_tweets' in tweet_obj['data'][i].keys():

try:

rftt = str(tweet_obj['data'][i]['referenced_tweets'][0]['type'])

except:

rftt = str('')

try:

rftid = str(tweet_obj['data'][i]['referenced_tweets'][0]['id']) + "g"

except:

rftid = str('')

if 'in_reply_to_user_id' in tweet_obj['data'][i].keys():

irtuid = str(tweet_obj['data'][i]['in_reply_to_user_id']) + "g"

if "public_metrics" in tweet_obj['data'][i].keys():

try:

retc = str(tweet_obj['data'][i]['public_metrics']['retweet_count'])

except:

retc = ''

try:

repc = str(tweet_obj['data'][i]['public_metrics']['reply_count'])

except:

repc = ''

try:

likc = str(tweet_obj['data'][i]['public_metrics']['like_count'])

except:

likc = ''

try:

quotc = str(tweet_obj['data'][i]['public_metrics']['quote_count'])

except:

quotc = ''

info.append(str(irtuid))

info.append(str(tl))

info.append(str(retc))

info.append(str(repc))

info.append(str(likc))

info.append(str(quotc))

info.append(str(rftt))

info.append(str(rftid))

info.append(str(src))

if len(finds)==1: #no need to check substring matches

pass

else: #remove matches that are proper subsets of other matches

trueFinds = copy.deepcopy(finds)

for find1 in finds:

if type(find1)==str:

continue

for find2 in finds:

if type(find2[0])==str:

continue

if find1 != find2 and find1[0]>=find2[0] and find1[1]<=find2[1]:

try:

trueFinds.remove(find1)

except:

print("couldn't remove: {}".format(find1))

continue

finds = trueFinds

totalFinds += len(finds)

#negativeSum = 0

raceCategories = []

raceTerms = []

#order is negativesum, then all race categories, then all race terms

for find in finds: #tuples represent start, end point in str

if type(find[0])==int:#from continuous string searches

s = tweet[find[0]:find[1]]

else:

s = find #from e.g. "tamir + rice"

#print("found: {}".format(s))

#negativeSum = negativeSum + raceDict[s]["Negative"]

raceCategories.append(raceDict[s]["Race"])

raceTerms.append(s)

#but even if there are fewer than 3 race terms, we must pad this list with empty string

while len(raceCategories)<3:

raceCategories.append('')

while len(raceTerms)<3:

raceTerms.append('')

#info.append(str(negativeSum))

#34. race cate 1

#35. race cate 2

#36. race cate 3

#37. race term 1

#38. race term 2

#39. race term 3

info.extend(raceCategories)

info.extend(raceTerms)

writer.writerow(info) #\t

if totalFinds%100==0:

OUTFILE.flush()

counts="Finished. Decode errors: {}; Value errors: {}; Type or Key errors = {}; No errors: {}".format(decodeErrors, valueErrors, typeKeyErrors, noErrors)

print(counts)

OUTFILE.close()

return(counts)

if __name__ == "__main__":

time0=time.time()

raceDict = processKeywords(infile)

processTweets(indir, outfile, raceDict)

time1=time.time()

process_time=time1-time0

print (process_time)

# code execution looks like:

# Finished. Decode errors: 0; Value errors: 0; Type or Key errors = 156; No errors: 1621660

# 250.12740087509155

# Finished. Decode errors: 0; Value errors: 0; Type or Key errors = 156; No errors: 1621660

# 2505.21435213089

**Step 3. Add FIPS Codes and Clean Locations (R Markdown)**

output: html_document

date: '2023-03-13'

---

#### load libraries

```{r}

library(tidyverse)

library(readr)

library(haven)

```

#### define target file

```{r}

# define the target file

tweets_df <- read_csv("E:/filtered_historical_tweets.csv")

```

```{r}

# see if there are duplicates

tweets_df <- tweets_df %>%

distinct()

```

#### clean location variables

* create new variables "city" and "state" based on the existing variable "place_full_name".

* correct some cases for these two new variables, for example, the city variable may have "Ohio" as its value.

```{r}

# may need to change

try(tweets_df$state <- str_extract(tweets_df$place_full_name,";.*"))

tweets_df$state <- gsub(";", "", tweets_df$state)

tweets_df$state <- gsub(" ", "", tweets_df$state)

```

```{r}

# may need to change

try(tweets_df$city <- str_extract(tweets_df$place_full_name,"[^;]*"))

```

```{r}

tweets_df <- tweets_df %>%

mutate(state = case_when(

city == "Georgia" ~ "GA",

city == "Florida" ~ "FL",

city == "Texas" ~ "TX",

city == "Pennsylvania" ~ "PA",

city == "Virginia" ~ "VA",

city == "North Carolina" ~ "NC",

city == "New York" ~ "NY",

city == "California" ~ "CA",

city == "South Carolina" ~ "SC",

city == "Colorado" ~ "CO",

city == "Illinois" ~ "IL",

city == "Kentucky" ~ "KY",

city == "Alabama" ~ "AL",

city == "Ohio" ~ "OH",

city == "Mississippi" ~ "MS",

city == "Tennessee" ~ "TN",

city == "Washington" ~ "DC",

city == "Arizona" ~ "AL",

city == "Nevada" ~ "NV",

city == "Louisiana" ~ "LA",

city == "Michigan" ~ "MI",

city == "New Jersey" ~ "NJ",

city == "Maryland" ~ "MD",

city == "Missouri" ~ "MO",

city == "Kansas" ~ "KS",

city == "Delaware" ~ "DE",

city == "Wisconsin" ~ "WI",

city == "Indiana" ~ "IN",

city == "Arkansas" ~ "AR",

city == "Minnesota" ~ "MN",

city == "Oklahoma" ~ "OK",

city == "Massachusetts" ~ "MA",

city == "Oregon" ~ "OR",

city == "Iowa" ~ "IA",

city == "West Virginia" ~ "WV",

city == "Connecticut" ~ "CT",

city == "New Mexico" ~ "NM",

city == "Maine" ~ "ME",

city == "Nebraska" ~ "NE",

city == "New Hampshire" ~ "NH",

city == "Idaho" ~ "ID",

city == "Utah" ~ "UT",

city == "Hawaii" ~ "HI",

city == "South Dakota" ~ "SD",

city == "Vermont" ~ "VT",

city == "Guam" ~ "GU",

city == "Montana" ~ "MT",

city == "North Dakota" ~ "ND",

city == "Northern Mariana Islands" ~ "CM",

city == "Puerto Rico" ~ "PR",

city == "Rhode Island" ~ "RI",

city == "Trust Territories" ~ "TT",

city == "Virgin Islands" ~ "VI",

city == "Wyoming" ~ "WY",

city == "District of Columbia" ~ "DC",

city == "Alaska" ~ "AK",

city == "Tumon" ~ "GU",

city == "Dededo" ~ "GU",

city == "Ordot" ~ "GU",

city == "Barrigada" ~ "GU",

city == "Barrigada Heights" ~ "GU",

city == "Maite" ~ "GU",

city == "Agana Heights" ~ "GU",

city == "Santa Rita" ~ "CA",

city == "Playa Sardinas I" ~ "PR",

TRUE ~ state

)) %>%

mutate(city = case_when(

city == "Georgia" ~ NA_character_,

city == "Manhattan" ~ "New York",

city == "Florida" ~ NA_character_,

city == "Texas" ~ NA_character_,

city == "Queens" ~ "New York",

city == "Pennsylvania" ~ NA_character_,

city == "Virginia" ~ NA_character_,

city == "North Carolina" ~ NA_character_,

city == "New York" ~ NA_character_,

city == "California" ~ NA_character_,

city == "South Carolina" ~ NA_character_,

#city == "Paradise" ~ NA_character_,

city == "Colorado" ~ NA_character_,

city == "Illinois" ~ NA_character_,

city == "Kentucky" ~ NA_character_,

city == "Alabama" ~ NA_character_,

city == "Ohio" ~ NA_character_,

city == "Mississippi" ~ NA_character_,

city == "Tennessee" ~ NA_character_,

city == "Washington" ~ NA_character_,

city == "Arizona" ~ NA_character_,

city == "Nevada" ~ NA_character_,

city == "Louisiana" ~ NA_character_,

city == "Michigan" ~ NA_character_,

city == "New Jersey" ~ NA_character_,

city == "Maryland" ~ NA_character_,

city == "Missouri" ~ NA_character_,

city == "Kansas" ~ NA_character_,

city == "Delaware" ~ NA_character_,

city == "Wisconsin" ~ NA_character_,

city == "Indiana" ~ NA_character_,

city == "Arkansas" ~ NA_character_,

city == "Minnesota" ~ NA_character_,

city == "Oklahoma" ~ NA_character_,

city == "Massachusetts" ~ NA_character_,

city == "Oregon" ~ NA_character_,

city == "Iowa" ~ NA_character_,

city == "West Virginia" ~ NA_character_,

#city == "Enterprise" ~ NA_character_,

city == "Connecticut" ~ NA_character_,

city == "New Mexico" ~ NA_character_,

city == "Maine" ~ NA_character_,

city == "Nebraska" ~ NA_character_,

city == "New Hampshire" ~ NA_character_,

#city == "University" ~ NA_character_,

city == "Idaho" ~ NA_character_,

city == "Utah" ~ NA_character_,

city == "Hawaii" ~ NA_character_,

city == "South Dakota" ~ NA_character_,

city == "Vermont" ~ NA_character_,

city == "Guam" ~ NA_character_,

city == "Montana" ~ NA_character_,

city == "North Dakota" ~ NA_character_,

city == "Northern Mariana Islands" ~ NA_character_,

city == "Puerto Rico" ~ NA_character_,

city == "Rhode Island" ~ NA_character_,

city == "Trust Territories" ~ NA_character_,

city == "Virgin Islands" ~ NA_character_,

city == "Wyoming" ~ NA_character_,

city == "District of Columbia" ~ NA_character_,

city == "Alaska" ~ NA_character_,

city == "Tumon" ~ NA_character_,

city == "Dededo" ~ NA_character_,

city == "Ordot" ~ NA_character_,

city == "Barrigada" ~ NA_character_,

city == "Barrigada Heights" ~ NA_character_,

city == "Maite" ~ NA_character_,

city == "Agana Heights" ~ NA_character_,

city == "Santa Rita" ~ NA_character_,

city == "Playa Sardinas I" ~ NA_character_,

TRUE ~ city

))

tweets_df

```

```{r}

tweets_df <- tweets_df %>%

mutate(state = case_when(state == "USA" ~ NA_character_,

place_full_name == "United States" | is.na(place_full_name) ~ NA_character_,

TRUE ~ state),

city = case_when(place_full_name == "United States" | is.na(place_full_name) ~ NA_character_,

TRUE ~ city))

```

```{r}

# may need to change if string format error occurs

tweets_df <- tweets_df %>%

mutate(city = case_when(city == "United States" ~ NA_character_,

TRUE ~ city)) %>%

mutate(place_b_check = case_when(is.na(state) & is.na(city) ~ 1,

TRUE ~ NA_real_)) %>%

mutate(state = case_when(place_b_check == 1 ~ str_extract(user_place,";.*"),

TRUE ~ state),

city = case_when(place_b_check == 1 ~ str_extract(user_place,"[^;]*"),

TRUE ~ city))

```

```{r}

# may need to change

tweets_df$state <- gsub(" ", "", tweets_df$state)

tweets_df$state <- gsub(";", "", tweets_df$state)

```

```{r}

tweets_df <- tweets_df %>%

mutate(city = case_when(city == "United States" ~ NA_character_,

TRUE ~ city)) %>%

mutate(state = case_when(

tolower(city) == "georgia" ~ "GA",

tolower(city) == "florida" ~ "FL",

tolower(city) == "texas" ~ "TX",

tolower(city) == "pennsylvania" ~ "PA",

tolower(city) == "virginia" ~ "VA",

tolower(city) == "north Carolina" ~ "NC",

tolower(city) == "new York" ~ "NY",

tolower(city) == "california" ~ "CA",

tolower(city) == "south Carolina" ~ "SC",

tolower(city) == "colorado" ~ "CO",

tolower(city) == "illinois" ~ "IL",

tolower(city) == "kentucky" ~ "KY",

tolower(city) == "alabama" ~ "AL",

tolower(city) == "ohio" ~ "OH",

tolower(city) == "mississippi" ~ "MS",

tolower(city) == "tennessee" ~ "TN",

tolower(city) == "washington" ~ "DC",

tolower(city) == "arizona" ~ "AL",

tolower(city) == "nevada" ~ "NV",

tolower(city) == "louisiana" ~ "LA",

tolower(city) == "michigan" ~ "MI",

tolower(city) == "new Jersey" ~ "NJ",

tolower(city) == "maryland" ~ "MD",

tolower(city) == "missouri" ~ "MO",

tolower(city) == "kansas" ~ "KS",

tolower(city) == "delaware" ~ "DE",

tolower(city) == "wisconsin" ~ "WI",

tolower(city) == "indiana" ~ "IN",

tolower(city) == "arkansas" ~ "AR",

tolower(city) == "minnesota" ~ "MN",

tolower(city) == "oklahoma" ~ "OK",

tolower(city) == "massachusetts" ~ "MA",

tolower(city) == "oregon" ~ "OR",

tolower(city) == "iowa" ~ "IA",

tolower(city) == "west Virginia" ~ "WV",

tolower(city) == "connecticut" ~ "CT",

tolower(city) == "new Mexico" ~ "NM",

tolower(city) == "maine" ~ "ME",

tolower(city) == "nebraska" ~ "NE",

tolower(city) == "new Hampshire" ~ "NH",

tolower(city) == "idaho" ~ "ID",

tolower(city) == "utah" ~ "UT",

tolower(city) == "hawaii" ~ "HI",

tolower(city) == "south Dakota" ~ "SD",

tolower(city) == "vermont" ~ "VT",

tolower(city) == "guam" ~ "GU",

tolower(city) == "montana" ~ "MT",

tolower(city) == "north Dakota" ~ "ND",

tolower(city) == "northern Mariana Islands" ~ "CM",

tolower(city) == "puerto Rico" ~ "PR",

tolower(city) == "rhode Island" ~ "RI",

tolower(city) == "trust Territories" ~ "TT",

tolower(city) == "virgin Islands" ~ "VI",

tolower(city) == "wyoming" ~ "WY",

tolower(city) == "district of Columbia" ~ "DC",

tolower(city) == "alaska" ~ "AK",

tolower(city) == "tumon" ~ "GU",

tolower(city) == "dededo" ~ "GU",

tolower(city) == "ordot" ~ "GU",

tolower(city) == "barrigada" ~ "GU",

tolower(city) == "barrigada Heights" ~ "GU",

tolower(city) == "maite" ~ "GU",

tolower(city) == "agana Heights" ~ "GU",

tolower(city) == "santa Rita" ~ "CA",

tolower(city) == "playa Sardinas I" ~ "PR",

TRUE ~ state

)) %>%

mutate(city = case_when(

tolower(city) == "georgia" ~ NA_character_,

tolower(city) == "manhattan" ~ "New York",

tolower(city) == "florida" ~ NA_character_,

tolower(city) == "texas" ~ NA_character_,

tolower(city) == "queens" ~ "New York",

tolower(city) == "pennsylvania" ~ NA_character_,

tolower(city) == "virginia" ~ NA_character_,

tolower(city) == "north Carolina" ~ NA_character_,

tolower(city) == "new York" ~ NA_character_,

tolower(city) == "california" ~ NA_character_,

tolower(city) == "south Carolina" ~ NA_character_,

#city == "Paradise" ~ NA_character_,

tolower(city) == "colorado" ~ NA_character_,

tolower(city) == "illinois" ~ NA_character_,

tolower(city) == "kentucky" ~ NA_character_,

tolower(city) == "alabama" ~ NA_character_,

tolower(city) == "ohio" ~ NA_character_,

tolower(city) == "mississippi" ~ NA_character_,

tolower(city) == "tennessee" ~ NA_character_,

tolower(city) == "washington" ~ NA_character_,

tolower(city) == "arizona" ~ NA_character_,

tolower(city) == "nevada" ~ NA_character_,

tolower(city) == "louisiana" ~ NA_character_,

tolower(city) == "michigan" ~ NA_character_,

tolower(city) == "new Jersey" ~ NA_character_,

tolower(city) == "maryland" ~ NA_character_,

tolower(city) == "missouri" ~ NA_character_,

tolower(city) == "kansas" ~ NA_character_,

tolower(city) == "delaware" ~ NA_character_,

tolower(city) == "wisconsin" ~ NA_character_,

tolower(city) == "indiana" ~ NA_character_,

tolower(city) == "arkansas" ~ NA_character_,

tolower(city) == "minnesota" ~ NA_character_,

tolower(city) == "oklahoma" ~ NA_character_,

tolower(city) == "massachusetts" ~ NA_character_,

tolower(city) == "oregon" ~ NA_character_,

tolower(city) == "iowa" ~ NA_character_,

tolower(city) == "west Virginia" ~ NA_character_,

#city == "Enterprise" ~ NA_character_,

tolower(city) == "connecticut" ~ NA_character_,

tolower(city) == "new Mexico" ~ NA_character_,

tolower(city) == "maine" ~ NA_character_,

tolower(city) == "nebraska" ~ NA_character_,

tolower(city) == "new Hampshire" ~ NA_character_,

#city == "University" ~ NA_character_,

tolower(city) == "idaho" ~ NA_character_,

tolower(city) == "utah" ~ NA_character_,

tolower(city) == "hawaii" ~ NA_character_,

tolower(city) == "south Dakota" ~ NA_character_,

tolower(city) == "vermont" ~ NA_character_,

tolower(city) == "guam" ~ NA_character_,

tolower(city) == "montana" ~ NA_character_,

tolower(city) == "north Dakota" ~ NA_character_,

tolower(city) == "northern Mariana Islands" ~ NA_character_,

tolower(city) == "puerto Rico" ~ NA_character_,

tolower(city) == "rhode Island" ~ NA_character_,

tolower(city) == "trust Territories" ~ NA_character_,

tolower(city) == "virgin Islands" ~ NA_character_,

tolower(city) == "wyoming" ~ NA_character_,

tolower(city) == "district of Columbia" ~ NA_character_,

tolower(city) == "alaska" ~ NA_character_,

tolower(city) == "tumon" ~ NA_character_,

tolower(city) == "dededo" ~ NA_character_,

tolower(city) == "ordot" ~ NA_character_,

tolower(city) == "barrigada" ~ NA_character_,

tolower(city) == "barrigada Heights" ~ NA_character_,

tolower(city) == "maite" ~ NA_character_,

tolower(city) == "agana Heights" ~ NA_character_,

tolower(city) == "santa Rita" ~ NA_character_,

tolower(city) == "playa Sardinas I" ~ NA_character_,

TRUE ~ city

))

```

```{r}

tweets_df <- tweets_df %>%

mutate(city = case_when(state == "Arlington" ~ "Arlington",

state == "Austin" ~ "Austin",

state == "AtlanticBeach" ~ "AtlanticBeach",

state == "Atlanta" ~ "Atlanta",

state == "Baltimore" ~ "Baltimore",

state == "Bellevue" ~ "Bellevue",

state == "Bexley" ~ "Bexley",

state == "Boston" ~ "Boston",

state == "Bronx" ~ "Bronx",

state == "Brooklyn" ~ "Brooklyn",

TRUE ~ city)) %>%

mutate(state = case_when(state == "Arlington" ~ "VA",

state == "Austin" ~ "TX",

state == "AtlanticBeach" ~ "NC",

state == "Atlanta" ~ "GA",

state == "Baltimore" ~ "MD",

state == "Bellevue" ~ "WA",

state == "Bexley" ~ "OH",

state == "Boston" ~ "MA",

state == "Bronx" ~ "NY",

state == "Brooklyn" ~ "NY",

TRUE ~ state))

```

```{r}

tweets_df <- tweets_df %>%

select(-c(place_b_check))

```

#### add state and county fips

```{r}

# reference files for state fips and county fips

statefips <- read_csv("E:/statefips.csv",

col_types = cols(state = col_skip()))

fipscodes <- read_csv("E:/fipscodes.csv")

```

```{r}

statefips <- statefips %>%

rename(state = stateinitials)

```

```{r}

tweets_df_state <- merge(tweets_df, statefips, all.x = TRUE)

tweets_df_state

```

```{r}

countyfips <- fipscodes %>%

select(state, countyfips, cityr2) %>%

rename(city = cityr2)

```

```{r}

tweets_df_county <- left_join(tweets_df_state, countyfips, by = c("city", "state"))

```

#### save result file

```{r}

write_csv(tweets_df_county, "E:/filtered_tweets_with_countyandstatefips.csv")

```

**Step 4: Sentiment Analysis Models (Python Programming Language)**

**Training Negative and Positive Sentiment Models**

# run the file "sentiment_prediction_example.py" first on your target file.

# if error shows telling you that there is a model version issue,

# you may need to run this file "sentiment_train_example.py" to update the model version.

#!/usr/bin/env python

# coding: utf-8

#classifier: SVM

#data used for training: sentiment140 mannual, sanders, kaggle, thu, qc

from time import time

import scipy

import numpy

import pandas as pd

import re

from sklearn.feature_extraction.text import CountVectorizer

import sys

import sklearn

import codecs

from sklearn.naive_bayes import MultinomialNB

from sklearn.feature_extraction.text import TfidfVectorizer

from sklearn.linear_model import LogisticRegression

from sklearn.linear_model import SGDClassifier

from sklearn.metrics import accuracy_score

from sklearn.metrics import confusion_matrix

from sklearn.metrics import f1_score

from sklearn.model_selection import GridSearchCV

from sklearn.utils import shuffle

import numpy as np

import pickle

import builtins

from sklearn.feature_extraction.text import CountVectorizer

from sklearn.feature_extraction.text import TfidfTransformer

from sklearn import linear_model

from sklearn.model_selection import StratifiedKFold

from sklearn.pipeline import Pipeline

from pprint import pprint

import nltk

nltk.download('stopwords')

from nltk.corpus import stopwords

stop=stopwords.words('english')

import pandas as pd

import numpy as np

import os

import joblib

os.chdir('E:\\twitter_project\\sentiment_model\\sentiment_model')

#check scikit-learn version

#if new version is installed on the user's computer

#the user may need to update the code a little bit

#since some functions may deprecate/replaced by new ones

import sklearn

sklearn.__version__

#######################################

###sad model start

#######################################

###read in data with labels from our group

thu=pd.read_csv('E:\\twitter_project\\sentiment_model\\sentiment_model\\training_data\\thu.csv')

thu=thu.dropna(axis=0, subset=['text','target','sad_manual'])

thu['sad_manual']=thu['sad_manual'].astype(np.int64)

thutext=thu['text'].tolist()

thutarget=thu['target'].tolist()

thusad=thu['sad_manual'].tolist()

thutextarray=np.array(thutext)

thusadarray=np.array(thusad)

thutargetarray=np.array(thutarget)

train_no_1401=pd.read_csv('E:\\twitter_project\\sentiment_model\\sentiment_model\\training_data\\train_no_1401.csv')

###define sadness: neutral and irrelevant are categorized as not sad(sad=0)

def definesad(train_no_1401):

if train_no_1401['target']==0:

return 1

else:

return 0

train_no_1401['sad']=train_no_1401.apply(lambda train_no_1401: definesad(train_no_1401), axis=1)

train_no_1401['sad']=train_no_1401['sad'].astype(np.int64)

train_no_1401=train_no_1401.dropna(axis=0, subset=['text','sad'])

train_sad_text=train_no_1401['text'].tolist()+thutext

train_sad_y=train_no_1401['sad'].tolist()+thusad

sadtextarray=np.array(train_sad_text)

sadyarray=np.array(train_sad_y)

### train sad model

#model selection is performed elsewhere

#after comparison, we decided to use linear SVM

#the choice of parameters within SVM does not affect the result too much

import joblib

from sklearn import svm

tvec = TfidfVectorizer(stop_words=stop)

model = svm.SVC(kernel='linear', C=1)

pipe = Pipeline([

('vectorizer', tvec),

('classifier', model)

])

sentiment_fit=pipe.fit(sadtextarray,sadyarray)

#dump model to pkl and saved

#next time the user just need to read in the models and then perform prediction

joblib.dump(model, 'E:\\twitter_project\\sentiment_model\\sentiment_model\\model_0.22.2.post1\\svm_sad.pkl')

joblib.dump(tvec, 'E:\\twitter_project\\sentiment_model\\sentiment_model\\model_0.22.2.post1\\svm_sad_vectorizer.pkl')

#######################################

###sad model end

#######################################

# test checking

s = ['I love u', 'i hate u','I dont like vegetable']

sentiment_fit.predict(s)

#######################################

###happy model start

#######################################

#first read in happy tweets

qc=pd.read_csv('E:\\twitter_project\\sentiment_model\\sentiment_model\\training_data\\qc.csv')

qc=qc.dropna(axis=0, how='any')

def definehappy(train_no_1401):

if train_no_1401['target']==1:

return 1

else:

return 0

train_no_1401['happy']=train_no_1401.apply(lambda train_no_1401: definehappy(train_no_1401), axis=1)

qc['happy']=qc.apply(lambda qc: definehappy(qc), axis=1)

thu['happy']=thu.apply(lambda thu: definehappy(thu), axis=1)

train_happy_text=train_no_1401['text'].tolist()+thu['text'].tolist()+qc['clean_text'].tolist()

train_happy_y=train_no_1401['happy'].tolist()+thu['happy'].tolist()+qc['happy'].tolist()

happytextarray=np.array(train_happy_text)

happyyarray=np.array(train_happy_y)

#train happy model

#from sklearn.externals import joblib

#import joblib

#from sklearn import svm

tvec = TfidfVectorizer(stop_words=stop)

model = svm.SVC(kernel='linear', C=1)

pipe = Pipeline([

('vectorizer', tvec),

('classifier', model)

])

happy_fit=pipe.fit(happytextarray,happyyarray)

#from sklearn.externals import joblib

joblib.dump(model, 'E:\\twitter_project\\sentiment_model\\sentiment_model\\model_0.22.2.post1\\svm_happy.pkl')

joblib.dump(tvec, 'E:\\twitter_project\\sentiment_model\\sentiment_model\\model_0.22.2.post1\\svm_happy_vectorizer.pkl')

#######################################

###happy model end

#######################################

#test checking

s = ['I love u', 'i hate u','I dont like vegetable']

happy_fit.predict(s)

**Step 5: Run sentiment predictions (Python Programming Language)**

import re

from bs4 import BeautifulSoup

import html5lib

import lxml

from nltk.tokenize import WordPunctTokenizer

# run codes here on your target file

# if error shows telling you that there is a model version issue,

# you may need to run the file "sentiment_train_example.py" to update the model version.

# to get both sad and happy sentiments, you will need to run this code twice.

# see the lines where I indicate, switch model between "happy" and "sad".

# clean the tweets

tok = WordPunctTokenizer()

pat1 = r'@[A-Za-z0-9_]+'

pat2 = r'https?://[^ ]+'

pat3 = r'\\'

pat4= r':\\'

emotion=r'[:;]+["^-]*[()]+'

combined_pat = r'|'.join((pat1, pat2, pat3, emotion))

www_pat = r'www.[^ ]+'

negations_dic = {"isn't":"is not", "aren't":"are not", "wasn't":"was not", "weren't":"were not",

"haven't":"have not","hasn't":"has not","hadn't":"had not","won't":"will not",

"wouldn't":"would not", "don't":"do not", "doesn't":"does not","didn't":"did not",

"can't":"can not","couldn't":"could not","shouldn't":"should not","mightn't":"might not",

"mustn't":"must not"}

neg_pattern = re.compile(r'\b(' + '|'.join(negations_dic.keys()) + r')\b')

def tweet_cleaner(text):

neg_handled = neg_pattern.sub(lambda x: negations_dic[x.group()], text)

soup = BeautifulSoup(neg_handled, 'html.parser')

souped = soup.get_text()

try:

bom_removed = souped.encode('ascii', 'ignore').decode('utf-8-sig').replace(u"\ufffd", "?")

except:

bom_removed = souped

stripped = re.sub(combined_pat, '', bom_removed)

stripped = re.sub(www_pat, '', stripped)

lower_case = stripped.lower()

letters_only = re.sub("[^a-zA-Z]", " ", lower_case)

# During the letters_only process two lines above, it has created unnecessay white spaces,

# I will tokenize and join together to remove unneccessary white spaces

words = [x for x in tok.tokenize(letters_only) if len(x) > 1]

return (" ".join(words)).strip()

import os

import glob

import pandas as pd

# from sklearn.externals import joblib

import joblib

from sklearn.pipeline import Pipeline

# pkl_path is the directory to save sentiment models

# we have both models to predict happy and sad,

# change "happy" to "sad" on where I indicate to change the model.

pkl_path="E:\\twitter_project\\sentiment_model\\sentiment_model\\model_0.22.2.post1\\"

m_file=pkl_path+'svm_happy.pkl' ############# sad model: m_file=pkl_path+'svm_sad.pkl'

vec_file=pkl_path+'svm_happy_vectorizer.pkl' ############### sad model: vec_file=pkl_path+'svm_sad_vectorizer.pkl'

model = joblib.load(m_file)

vec = joblib.load(vec_file)

text_clf = Pipeline([('vect', vec),

('clf', model),

])

# path is the directory that saves all the processed tweet files

# code will process all files that saved under this path

path="E:\\sent\\"

path2 = "E:\\sent\\"

for file in os.listdir(path):

filepath=os.path.join(path, file)

rawtweet=pd.read_csv(filepath, error_bad_lines=False, encoding='utf8') #,sep="\t"

# encoding='utf8'

cleaned_tweet=[]

sad=[]

for t in rawtweet['tweet_text']: #tweet_text

cleaned_tweet.append(tweet_cleaner(t))

rawtweet['tweet_id']=rawtweet['tweet_id']

rawtweet['cleaned_tweet']=cleaned_tweet

sad=text_clf.predict(cleaned_tweet)

rawtweet['happy']=sad ################ dont forget to change csv variable name. sad model: rawtweet['sad']=sad

# save output file

rawtweet.to_csv(path2+ file + 'happy.csv') ############# dont forget to change csv name. sad model: rawtweet.to_csv(path2+ file + 'sad.csv')

**Step 6: Sentiment Analysis with Birth Outcomes (Stata)**

/** sentiment variables ***/

gen blackcat = 1 if racecat1 == "black"

gen hispcat1 = 1 if (racecat1 == "hispanic" | racecat1 == "mexican")

gen asiancat = 1 if (racecat1 == "asian" | racecat1 == "pacific islander")

gen whitecat=0

replace whitecat=1 if (racecat1 == "white")

gen sentsadrace = sad

*add sad race sentiment without whites*;

gen sentsadrace_nowhite = sad if whitecat==0

gen sentsadwhite = sad if whitecat==1

gen sentsadblack = sad if blackcat==1

gen sentsadhisp1 = sad if hispcat1==1

gen sentsadasian = sad if asiancat==1

gen sentrace = happy

gen sentrace_nowhite = happy if whitecat==0

gen sentwhite = happy if whitecat==1

gen sentblack = happy if blackcat==1

gen senthisp1 = happy if hispcat1==1

gen sentasian = happy if asiancat==1

* remove 1000+ tweeters and save ===============

sort user_username

bysort user_username: gen totalcountweets=_N

keep if totalcountweets<1000

keep if statefp!=.

* save after removing 1000+ tweeters

* collapse by state and calculate tertiles and save ===============

collapse (mean) sentsad* year, by(statefp)

*next create tertiles*

xtile tertsentsadraces = sentsadrace, nquantiles(3)

xtile tertsentsadraces_nowhite = sentsadrace_nowhite, nquantiles(3)

xtile tertsentsadwhites = sentsadwhite, nquantiles(3)

xtile tertsentsadblacks = sentsadblack, nquantiles(3)

xtile tertsentsadmiddles = sentsadmiddle1, nquantiles(3)

xtile tertsentsadhisps = sentsadhisp1, nquantiles(3)

xtile tertsentsadasians = sentsadasian, nquantiles(3)

xtile tertsentsadjewishs = sentsadjewish, nquantiles(3)

* save after collapsing at state level

*create variables for analysis in birth outcomes data*

/*Birth outcomes*/

gen birthwt = dbwt

replace birthwt = . if dbwt == 9999

*birth weight*;

gen lbw = 1 if (birthwt <= 2499 & birthwt != .)

replace lbw = 0 if birthwt > 2499

replace lbw = . if birthwt == .

*measure to use for preterm*;

*<37 weeks*

generate pretermoegest=0

replace pretermoegest=1 if oegest_r3==1

* merge birth files with file created in first step

merge 1:m statefp year using [data set with state-level Twitter and American Community Survey data]

*Full sample*

glm lbw ib1.tertsentsadraces_nowhite i.year pblack_st phisp_st popdens_st econf south magesp* nhblack nhasian hispanic lhs somecollege college gradschool usborn married firstbirth firstpnc if (dplural==1 & nocongen==1), fam(bin) link(log) vce(cluster statefp) eform

*Racial sentiment referencing minoritized groups and birth outcomes among minoritized groups*

glm lbw ib1.tertsentsadraces_nowhite i.year pblack_st phisp_st popdens_st econf south magesp* nhblack nhasian hispanic lhs somecollege college gradschool usborn married firstbirth firstpnc if (dplural==1 & nocongen==1 & (hispanic==1 | usborn==0 | nhwhite==0)), fam(bin) link(log) vce(cluster statefp) eform

*Racial sentiment referencing minoritized groups and birth outcomes among White mothers*

glm lbw ib1.tertsentsadraces_nowhite i.year pblack_st phisp_st popdens_st econf south magesp* nhblack nhasian hispanic lhs somecollege college gradschool usborn married firstbirth firstpnc if (dplural==1 & nocongen==1 & nhwhite==1), fam(bin) link(log) vce(cluster statefp) eform

*Racial sentiment referencing Blacks and birth outcomes among Black mothers*

glm lbw ib1.tertsentsadblacks i.year pblack_st phisp_st popdens_st econf south magesp* nhblack nhasian hispanic lhs somecollege college gradschool usborn married firstbirth firstpnc if (dplural==1 & nocongen==1 & nhblack==1), fam(bin) link(log) vce(cluster statefp) eform

*Racial sentiment referencing Latinx and birth outcomes among Latina mothers*

glm lbw ib1.tertsentsadhisps i.year pblack_st phisp_st popdens_st econf south magesp* nhblack nhasian hispanic lhs somecollege college gradschool usborn married firstbirth firstpnc if (dplural==1 & nocongen==1 & hispanic==1), fam(bin) link(log) vce(cluster statefp) eform

*Racial sentiment referencing Latinx and birth outcomes among Latina mothers*

glm lbw ib1.tertsentsadasians i.year pblack_st phisp_st popdens_st econf south magesp* nhblack nhasian hispanic lhs somecollege college gradschool usborn married firstbirth firstpnc if (dplural==1 & nocongen==1 & nhasian==1), fam(bin) link(log) vce(cluster statefp) eform

*Run same models above, but now for preterm birth as the outcome
